# Jellyfish-Inspired Ultrafast and Versatile Magnetic Soft Robots for Biomedical Applications

**DOI:** 10.34133/cbsystems.0540

**Published:** 2026-04-03

**Authors:** Yuxuan Sun, Ruiqi Liu, Chiyuan Ma, Jingyang Liu, Semina Yi, Junnan Gu, Liangyu Xia, Haitao Qing, Kailin Cai, Liang Li, Lining Yao, Quanliang Cao

**Affiliations:** ^1^ Wuhan National High Magnetic Field Center, Huazhong University of Science and Technology, Wuhan 430074, China.; ^2^ School of Electrical and Electronic Engineering, Huazhong University of Science and Technology, Wuhan 430074, China.; ^3^ Department of Mechanical Engineering, University of California, Berkeley, Berkeley, CA 94704, USA.; ^4^ Department of Thoracic Surgery, Union Hospital, Tongji Medical College, Huazhong University of Science and Technology, Wuhan 430022, China.; ^5^ Department of Gastrointestinal Surgery, Union Hospital, Tongji Medical College, Huazhong University of Science and Technology, Wuhan 430022, China.

## Abstract

Achieving rapid and adaptive locomotion in soft robots is essential for navigating complex environments and enabling diverse real-world functions. Here, we present a jellyfish-inspired magnetic soft robot (J-MSR) capable of ultrafast swimming and seamless multimodal motion transitions in liquid environments. By employing an asymmetric trapezoidal magnetic field waveform for actuation, the J-MSR capitalizes on spatial and temporal asymmetries during its swimming cycle, mimicking the natural propulsion mechanism of jellyfish. Through magnetic-fluid-solid multiphysical field coupling analysis and magnetic field waveform optimization, the J-MSR achieves a remarkable swimming speed of 14.85 body lengths per second, demonstrating notably enhanced propulsion performance compared with previously reported jellyfish-inspired robots. Unlike traditional designs relying on auxiliary buoyancy structures, the J-MSR demonstrates versatile multimodal motions under natural negative buoyancy conditions, including large-angle multidirectional swimming (0° to 122°), slit traversal, and rolling. Meanwhile, its exceptional locomotion capabilities facilitate the integration of functional devices, enabling it to perform diverse tasks such as emitting light to mimic fluorescent jellyfish, capturing objects, injecting microneedles, and conducting gastroscopy. These capabilities highlight the J-MSR’s substantial potential as a versatile platform for biomedical applications in confined and unstructured environments.

## Introduction

Inspired by biological strategies for adaptation in complex environments, bioinspired design has become a powerful paradigm in soft robotics, enabling compliant, adaptive, and robust interactions in unstructured settings [1–4]. Moreover, recent advances in stimuli-responsive soft materials have further accelerated the development of intelligent and high-performance biomimetic soft robotic systems [5–7].

In nature, jellyfish, as soft-bodied animals with exceptional swimming efficiency, serve as ideal models for exploring the hydrodynamic principles of animal propulsion and advancing biomimetic engineering [8]. Building on these biological insights and leveraging stimuli-responsive actuation strategies, a variety of jellyfish-inspired robots have been developed, utilizing technologies such as dielectric elastic actuators [9–14], shape memory alloys [15,16], and light [17], or magnetic fields [18–23]. However, despite the promising potential of biological inspiration, the multi-degree-of-freedom deformation characteristics of soft components in jellyfish-inspired robots present both advantages and challenges in achieving high performance. Specifically, controlling soft-body deformation, especially for multidirectional motion, introduces considerable complexity, limiting efficient swimming and precise 3-dimensional (3D) trajectory control. Consequently, while notable progress has been made in jellyfish-inspired robotics, particularly in miniature robots, many designs are still confined to slow or monotonous motion [6,24], thereby restricting the research and practical applications of these bionic robots.

**Fig. 1.**
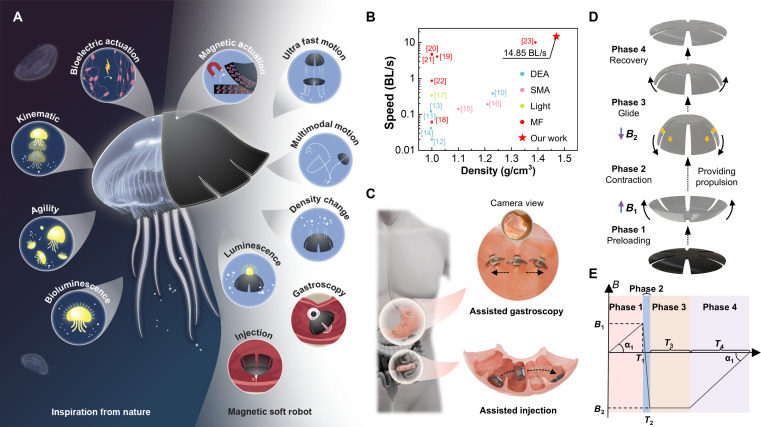
Robot design and actuation principles. (A) Bioinspiration from natural jellyfish and multifunctional capabilities of the jellyfish-inspired magnetic soft robot (J-MSR) platform. (B) Comparison of the speed and the density between our J-MSRs and other jellyfish-inspired robots reported in the literature [10–23]. The proposed J-MSR achieved a maximum speed of 14.85 BL/s (body lengths per second) while exhibiting a marked density difference with the liquid environment. (C) Biomedical applications of the J-MSRs. (D) Schematic illustration of the J-MSR’s swimming process under the actuation of the asymmetric trapezoidal magnetic field waveform. (E) Schematic of the asymmetric trapezoidal magnetic field waveform, highlighting key variables including the maximum positive magnetic flux density *B*_1_, the maximum negative magnetic flux density *B*_2_, the duration of the preload phase *T*_1_, the duration of the contraction phase *T*_2_, the duration of the glide phase *T*_3_, and the duration of the recovery phase *T*_4_.

To overcome these challenges, we developed a jellyfish-inspired magnetic soft robot (J-MSR) with a simple design yet exceptional performance (Fig. 1A). The adoption of magnetic field-responsive actuation was driven by its ability to enable remote, noncontact control, eliminate the need for an onboard power source, and leverage the versatile spatiotemporal characteristics of magnetic fields [25–28]. These attributes make it particularly advantageous in confined environments, miniaturized designs, and multimodal control applications, providing crucial support for overcoming the challenges. Existing jellyfish-like robots typically employ auxiliary buoyancy structures to maintain neutral buoyancy and enhance underwater motion performance [11–14,17–20]. However, such designs introduce additional drag and increase energy consumption during high-speed motion [29], while their low spatial efficiency constrains overall scalability and functional integration. In contrast, our J-MSR leverages magnetic field waveform modulation to greatly enhance propulsion performance, achieving an impressive swimming speed of 14.85 body lengths per second (BL/s) despite substantial density differences, thereby outperforming existing jellyfish robots (Fig. 1B). Here, 1 body length is defined as the diameter of the jellyfish-inspired robot in its fully expanded configuration. Our negative buoyancy design enables the J-MSR’s dynamic transitions across multiple locomotion modes in liquid environments. Its remarkable mobility and compact structure further support the integration of functional modules, including lighting, capturing, injecting, and performing gastroscopy (Fig. 1C), making it suitable for diverse operational scenarios in bodily cavities or lumens. Natural jellyfish swimming patterns demonstrate that their larger size and distinctive locomotion lead to operation at moderate to high Reynolds number ranges, where inertia becomes a dominant factor [30]. Their propulsion mechanism is based on temporal and spatial asymmetries. Similarly, our J-MSR emulates this behavior, with its swimming process comprising 4 phases (Fig. 1D), actuated by the asymmetric trapezoidal magnetic field waveform that we developed (Fig. 1E). During the contraction phase, the flexural deformation of the J-MSR’s lappets displaces a larger volume of fluid than during the preload phase, resulting in a greater swept area in the former. This disparity in swept areas between the 2 phases introduces “spatial asymmetry”, which ultimately propels the J-MSR forward [31]. Additionally, the introduction of a glide phase fully leverages the inertial effects of the robot’s kinematics, amplifying the “temporal asymmetry” across the phases. These 2 asymmetric effects directly determine the swimming performance of jellyfish robots. To analyze their contributions, we developed a speed-testing platform and a multiphysics coupling numerical analysis method encompassing magnetic-fluid-solid interactions in this work. Based on this, we quantitatively evaluated the effects of the 2 asymmetric phenomena on the robot’s swimming behavior by parameterizing the driving magnetic field waveform, thereby optimizing its performance. Furthermore, we constructed a 3D manipulation platform and a multifrequency magnetic actuation system, expanding the locomotion modalities and functional capabilities of the J-MSR. More details of our J-MSR and its comparison with existing jellyfish-inspired robotic work can be found in Table S1. Compared with previously reported platforms, the J-MSR achieves a maximum swimming speed of 14.85 BL/s, whereas the fastest speed reported in prior studies is approximately 10 BL/s. In terms of actuation strategy, our work introduces a 6-parameter waveform optimization framework, while most existing studies primarily focus on tuning only the driving frequency and amplitude. From a modeling perspective, we adopt a fully coupled magneto–solid–fluid simulation approach, whereas previous works are largely limited to single-field or 2-field coupling. Moreover, the proposed J-MSR demonstrates 3 distinct locomotion modes together with multiple functional demonstrations, including crossing narrow slits, object transport via a phase-change mechanism, and integration with a capsule endoscope. Collectively, these results highlight the enhanced versatility and overall performance of the proposed robot compared with existing jellyfish-inspired systems.

## Methods

### Bioinspired magnetostructural design of the J-MSRs

The design of the J-MSRs is inspired by *Aurelia aurita*, as shown in Fig. S1. Through biological analysis of *Aurelia aurita* [32], the structure of their muscle and nervous tissues is revealed, which serves as the foundation for their flexible swimming capability [33]. By replacing the corresponding biological muscle tissues with specific internal magnetization patterns and simulating neural stimulation signals via an external magnetic field, the J-MSRs can be obtained through tailored molding and magnetization processes (Fig. S2 and S3). More details can be found at the “Fabrication of the J-MSRs” section. In addition, the mechanical characteristics of the J-MSRs are presented in Fig. S4 and further described at the “Mechanical properties of the J-MSR” section. The robot is equipped with 6 soft lappets, with a minimum overall size of 10 mm. The ratio of the inner to outer diameter is designed as 1:1.8 to ensure optimal actuation capability [34], while the internal-to-external width ratio of the lappets is 0.44:1 (Note S1). This 6-lappet robot, when it contracts, forms a complete bell-shaped structure without gaps between the lappets, ensuring that all water is expelled downward. This design substantially enhances the motion capability of the J-MSRs. Furthermore, the lappets exhibit neither excessive width nor a propensity to overlap, thereby enhancing stability during movement. In addition, to enable the integration of multiple functional modules while accommodating the size requirements of representative medical devices (e.g., capsule endoscopes with diameters of approximately 10 to 12 mm), we further developed an expandable version of the J-MSRs, scaled up by a factor of 3.35. As a result, the individual lappets preserve the same length-to-thickness ratio before and after scaling, leading to similar deformation mechanics. Meanwhile, the central region of the robot was designed to incorporate a circular cavity with a diameter of 10 mm, which matches the characteristic size of commonly used capsule endoscopy systems. The sizes of different J-MSRs are shown in Fig. S5.

### Fabrication of the J-MSRs

The base material of the J-MSRs is a silicone elastomer (Ecoflex 00-10, Smooth-On Inc., United States), while the incorporated magnetic material is NdFeB powder (MQP-15-7, Tianjin Magnequench Co., Ltd., China). The entire fabrication process is illustrated in Fig. S2. First, NdFeB powder and Ecoflex 00-10 polymer are preliminarily mixed at a mass ratio of 1:2. The mixture is then placed in a planetary mixer (Thinky Corporation, Japan) and stirred at 2,000 r/min for 1 min to ensure thorough mixing of the hard magnetic particles with the soft polymer base. Following this, the mixture is centrifuged at 2,200 r/min for 45 s to remove air bubbles introduced during mixing. In the second step, the mixture is cast onto a flat acrylic plate with circular grooves 0.4 mm deep, forming the base of the J-MSRs. After approximately 4 h, the mixture naturally cools to room temperature, and the molded material is demolded using tweezers. The third step involves placing the preliminarily molded base material under a laser cutting machine (LMF50, China Optoelectronic Technology Co., Ltd., China) and cutting it with a 50-W laser. Finally, the laser-cut robot is magnetized using a mold-assisted magnetization method. As shown in Fig. S3, the magnetization system consists of a driving circuit and a magnetization coil. By controlling the on-off state of the diode freewheeling circuit, the coil is charged and discharged, generating a pulsed magnetic field for magnetizing the soft robot. The soft robot is magnetized under a 2-T background field while being held in a downward contraction state, resembling a bell shape. Finally, the resulting robot exhibits a density of 1.47 g/cm^3^ and a magnetization strength of 36.17 kA/m.

### Mechanical properties of the J-MSR

The mechanical properties of the composite soft material used to fabricate the J-MSR were characterized through uniaxial tensile testing. Experiments were conducted using a universal testing machine (MTS C43.504, MTS Systems Corporation, USA) equipped with a noncontact optical extensometer (VIC-2D, Beijing RealTect Innovation Technology Co., Ltd.), as illustrated in Fig. S4A. All specimens were tested at a constant loading rate of 100 mm/min. Standard type-2 dumbbell-shaped samples were prepared in accordance with GB/T 528–2009, and the corresponding geometric dimensions are shown in Fig. S4B.

Representative stress–strain curves obtained from the tensile tests are presented in Fig. S4C. The Young’s modulus *E* was extracted from the initial linear regime of the stress–strain response (*ε* < 0.3) and determined to be 46.08 kPa. Assuming a nearly incompressible elastomeric matrix, the corresponding shear modulus *G* was estimated using the relation *G* = *E*/3, yielding a value of 15.36 kPa.

### Hardware and software of 1-dimensional test platform

The 1-dimensional test platform comprises 2 main components: an electromagnetic drive system and a vision measurement system.

The hardware of the electromagnetic drive system consists of the control unit and the drive unit (as shown in Fig. S6). The control unit is responsible for receiving external signals, providing feedback, analyzing and processing data, and outputting low-voltage analog signals. The drive unit receives low-voltage control signals from the control unit, converts them into high-voltage drive signals, and precisely generates the corresponding spatial magnetic field to actuate the robots. The control unit is centered around a microcontroller (STM32F407ZGT6) and integrates peripheral modules, including a liquid-crystal display capacitive touchscreen (Guangzhou Xingyi Electronic Technology Co., Ltd, China), a universal asynchronous receiver-transmitter (UART) serial port, a PlayStation 2 (PS2) control handle, wireless switches and buttons, and a digital-to-analog converter module (DAC8563). The drive unit consists of a power amplifier and Helmholtz coils. A power amplifier (LYB-8050, Nantong Longyi Technology Co., Ltd., China) is used as the primary amplification component. This amplifier has a maximum output power of 4,000 W, a peak output current of 50 A, and an operational frequency range of 0 to 10 kHz. Compared with the previous 3 amplifiers, the LYB-8050 offers higher drive power and current output, enabling it to generate stronger unidirectional uniform magnetic fields through the Helmholtz coils. This provides a better parameter space for exploring unidirectional motion in magnetically controlled soft robots. Another set of coils used is a custom-designed single-axis Helmholtz coil (Hunan Yangyi Technology Co., Ltd., China). Based on the hardware, the developed software provides the following functionalities: (a) Reading input from external controllers, including button presses and joystick angles of the PS2 control handle; (b) accessing onboard memory codes; and (c) accepting user-defined parameters for target waveforms via a host computer, including amplitude, frequency, phase, and offset, and displaying these parameters on a monitor. It processes, calculates, and converts the input to generate the desired drive waveform. The touchscreen allows users to select the output waveform, after which the software transmits the waveform data to the DAC module. The DAC module outputs 3 control signals simultaneously and supports direct control of the waveform signal output through buttons or remote wireless switches.

The vision measurement system: The hardware component consists of an industrial camera, while the software leverages Qt and TensorRT for development. Qt serves as the user interface and is used to develop the camera driver in C++. Image data captured by the camera is processed through the target detection algorithm, with TensorRT as the inference backend. The selected target detection algorithm is YOLOv8 (You Only Look Once), which transforms the object detection problem into a regression task, enabling the prediction of object classes and locations within an image through a single forward pass. After identifying the robot’s position in the image, the data is transmitted via a serial port to the lower control system (STM32F407ZGT6).

### Multiphysics coupling simulation of the J-MSRs

This section presents the electromagnetic constitutive relations, the constitutive equations for solid materials, and the constitutive relations in flow fields, along with the multiphysics coupling mechanisms that integrate these domains. Additionally, it outlines the numerical simulation processes used to solve the coupled systems.

Electromagnetic constitutive relations: the interaction between a mass point of the robot and the external magnetic field can be characterized by the magnetic torque (***τ***), expressed as:

$$\tau =Μ\times B=\left|\begin{array}{ccc} i & j & k\\ M_{x} & M_{y} & M_{z}\\ B_{x} & B_{y} & B_{z} \end{array}\right|$$ (1)

where ***M*** is the magnetization at the mass point and ***B*** is the external magnetic flux density. In a 2-dimensional (2D) coordinate system where the z-component (*M*_*z*_, *B*_*z*_) is zero, the robot is divided into *n* equivalent elements. The distributed magnetic torque can then be represented as the cumulative magnetic torque, which is effectively calculated based on 2 tangential surface forces *F_n_* (N/m^2^) relative to the boundary frame, following the method proposed by Wu et al. [35]

$$F_{n}=M_{x}^{n}B_{y}-M_{y}^{n}B_{x}$$ (2)

where $M_{x}^{n}$ and $M_{y}^{n}$ represent the average magnetization of the element along the *x*_g-_ and *y*_g-_ axes, respectively, while *B*_*x*_ and *B*_*y*_ are the components of the magnetic flux density magnitude *B* in the *x*_g-_ and *y*_g-_ directions.

Constitutive relations in soft materials: Given the negligible effect of material compression due to magnetic field forces during deformation [36], the material is assumed to be incompressible to simplify computation when evaluating the mechanical stress component (*σ*_mech_) in hyperelastic materials. For incompressible hyperelastic materials, the Neo-Hookean material model, predefined in the COMSOL software, is employed. The strain energy density is expressed in terms of the elastic volume ratio *J*_el_ and the elastic right Cauchy–Green deformation tensor *I*_1_.

$$W_{s}=\frac{1}{2}\mu \left(I_{1}-3\right)-\mu \;\ln\left(J_{\mathrm{el}}\right)+\frac{1}{2}\lambda \;\ln\;{\left(J_{\mathrm{el}}\right)}^{2}$$ (3)

In this formula, *λ* represents the second Lamé parameter, which is correlated with the volumetric modulus of the material, and *μ* denotes the first Lamé parameter. The coupled strain energy density is determined based on these Lamé parameters, reflecting the material’s response to both volumetric and shear deformations.

$$W_{s}=\frac{1}{2}\mu \left(I_{1}-3\right)-\frac{\mu }{\beta }\left(J_{\mathrm{el}}^{-\beta }-1\right)$$ (4)

$$\beta =\frac{\lambda }{\mu }=\frac{2\nu }{1-2\nu }$$ (5)

Eq. (6) defines the value of *β* in Eq. (4). Here, *ν* represents Poisson’s ratio, which quantifies the relationship between lateral contraction and axial elongation of the material under mechanical stress.

Constitutive relations in flow fields: The flow field surrounding the robot is unsteady and nonuniform. Therefore, the full Navier–Stokes equations, incorporating inertial, convection, pressure, and diffusion terms, are solved. The fluid is assumed to be Newtonian and incompressible. Within this framework, the continuity equation ensures mass conservation:

$$\frac{\partial p_{f}}{\partial t}+\nabla \cdot \left(\rho u_{f}\right)=0$$ (6)

Here, *p*_f_ represents the pressure of the fluid, *ρ* denotes the fluid density, and ***u***_f_ stands for the fluid velocity vector. The vector equation that signifies momentum conservation:

$$\rho \frac{\partial u_{f}}{\partial t}+\rho \left(u_{f}\cdot \nabla \right)u_{f}=\nabla \cdot \left[-p_{f}I+K\right]+f$$ (7)

The viscous stress tensor, represented by ***K***, is a critical element within this model, expressed as:

$$K=\left(\mu_{f}\left(\nabla u_{f}+{\left(\nabla u_{f}\right)}^{T}\right)-\frac{2}{3}\mu_{f}\left(\nabla \cdot u_{f}\right)I\right)$$ (8)

where *μ*_f_ symbolizes the dynamic viscosity of the fluid.

Multiphysics coupling relationships: The interaction between the deformation structure (Material 1, modeled as a magnetic incompressible hyperelastic material) and the surrounding environmental medium (Material 2, composed of nonmagnetic substances like water) can be characterized by the following equation:

$$\nabla \cdot \left(\sigma_{2}-\sigma_{1}\right)=f=\rho_{\text{solid}}\frac{\partial u_{\text{solid}}}{\partial t}$$ (9)

This formula represents the exchange of momentum between the fluid and the solid. It can be interpreted as the force exerted by the fluid on the solid (denoted as ***f*** ) at the interface between them, leading to the accelerated motion of the solid. In the formula, *ρ*_solid_ signifies the density of the solid, while ***u***_solid_ denotes the velocity of the solid.

Numerical simulation approach: To model the deformation and motion characteristics of the J-MSRs in a liquid environment, a 2D finite element model (Fig. S7) was developed in COMSOL, incorporating the Magnetic Fields-No Current (MFNC), Solid Mechanics (Solid), and Laminar Flow (SPF) modules. To address nonlinear large deformations affecting the geometric structure, the arbitrary Lagrangian–Eulerian dynamic mesh method was implemented. This approach prevents mesh distortion and computational failures, ensuring numerical stability and accuracy. By dynamically adapting the mesh as the structure deforms, the arbitrary Lagrangian–Eulerian method enables real-time simulation of complex interactions among magnetic forces, fluid dynamics, and structural deformation. Note that, although the results from the simplified 2D simulation model exhibit some numerical differences compared to the experimental results (3D), the overall trends are highly similar, making the model suitable for analysis.

### 3D manipulation platform setup

This platform (Fig. S6B) differs from the 1D test platform primarily in its drive unit, which consists of 3 power amplifiers with different specifications (HEA series, Nanjing Forner Technology Co., China) and a 3D Helmholtz coil system (3HLY10-100, Changchun Yingpu Magnetic Electric Technology Co., China). The HEAS-50 power amplifier drives the z axis of the 3-axis Helmholtz coil, with a maximum output power of 200 W and a maximum output current of 3 A. The HEA-200C power amplifier is responsible for driving the y-axis coil, with a maximum output power of 550 W and a maximum output current of 30 A. The HEA-500G power amplifier drives the x-axis coil, with a maximum output power of 1,600 W and a maximum output current of 40 A. The 3HLY10-100 system comprises 3 coils, with the x-axis coil having an inner diameter of 393 mm, the y-axis coil having an inner diameter of 264 mm, and the z-axis coil having an inner diameter of 156 mm. Each coil is capable of generating a maximum uniform magnetic induction intensity of 15 mT within its effective inner diameter, and the uniformity within the effective region can reach 1% ± 0.2%.

## Results

### Actuation strategy and propulsion performance of the J-MSRs

As schematically shown in Fig. 1D, similar to the swimming process of jellyfish, the motion of the developed J-MSR in this work can be divided into 4 phases: preload, contraction, glide, and recovery. These phases are driven by an asymmetric trapezoidal magnetic field waveform (Fig. 1E). In contrast to commonly used sinusoidal or triangular waveforms shown in Table S1, the trapezoidal waveform incorporates more variables in this study, including 6 key parameters: the maximum positive magnetic flux density *B*_1_, the maximum negative magnetic flux density *B*_2_, the duration of the preload phase *T*_1_, the duration of the contraction phase *T*_2_, the duration of the glide phase *T*_3_, and the duration of the recovery phase *T*_4_. The inclusion of these multiple parameters provides increased flexibility in the design of locomotion performance for the J-MSR.

**Fig. 2.**
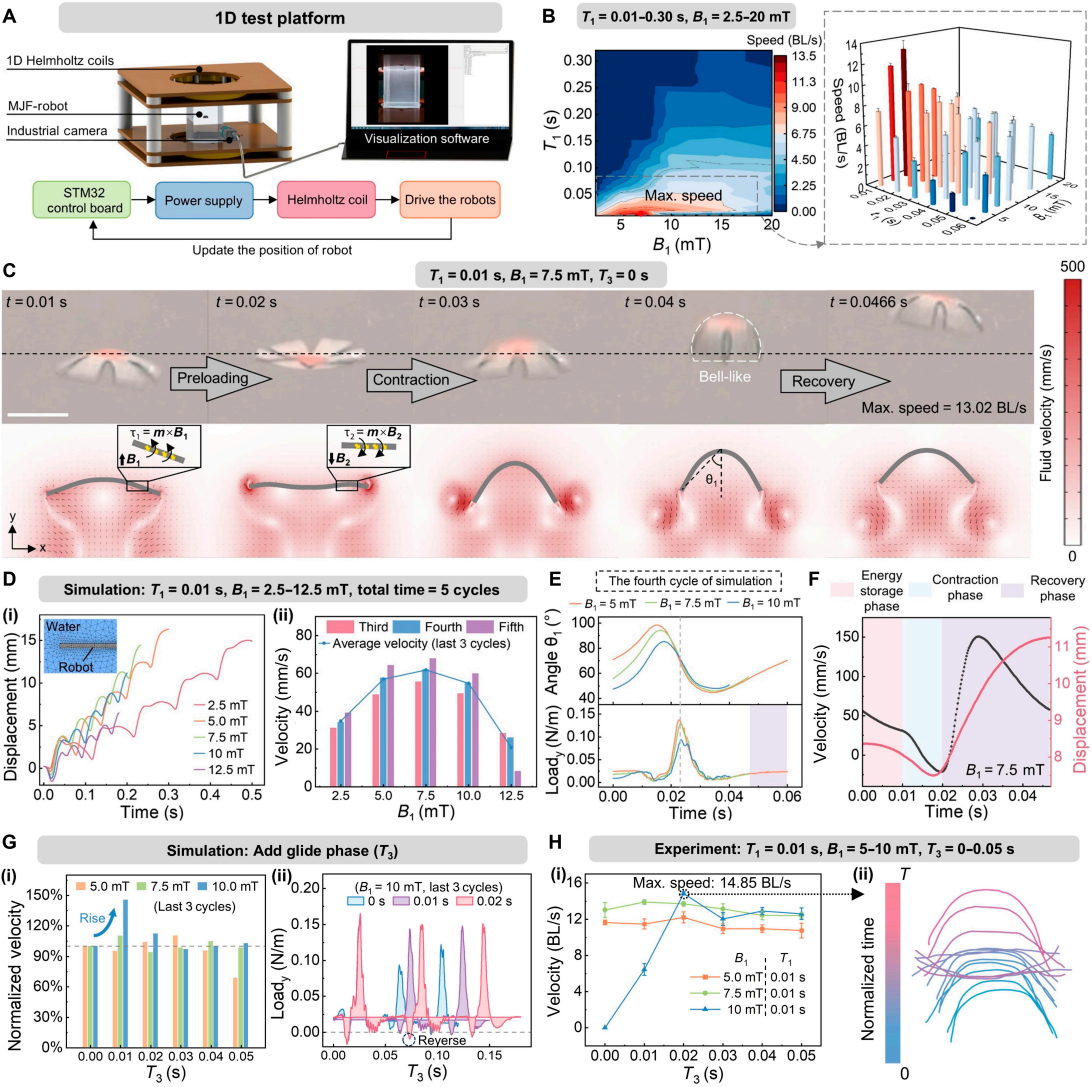
Experimental and simulation-based analysis and optimization of the jellyfish-inspired magnetic soft robot’s (J-MSR’s) vertical propulsion performance. (A) Schematic diagram of the developed 1-dimensional (1D) test platform, including an electromagnetic actuation system and a vision measurement system. (B) Speed variation trends of the J-MSRs within the ranges of *T*_1_ = 0.01 to 0.3 s and *B*_1_ = 2.5 to 20 mT (*T*_2_ = 0.01 s, *B*_2_ = −20 mT, *T*_3_ = 0 s, *T*_4_ = *T*_1_ × *B*_2_/*B*_1_, *n* = 3, data are presented as means ± SD). (C) Sequential motion diagrams of the robot in both experimental and simulation scenarios under conditions of *T*_1_ = 0.01 s, *B*_1_ = 7.5 mT, and *T*_3_ = 0 s. The simulation results also include flow field distribution diagrams. (D) Simulation analysis of the J-MSR under different *B*_1_ values, including displacement over 5 cycles (i) and velocity with average velocity over the last 3 cycles (ii). (E) During the fourth cycle in simulation, the bending angle θ_1_ of the robot’s lappets (as annotated in [C]) and the corresponding load in the y-direction, with the yellow area indicating the additional recovery time required when *B*_1_ = 2.5 mT. (F) Simulated nodal velocity and displacement profiles of J-MSR when *B*_1_ = 7.5 mT. (G) Simulation results with the introduction of the glide phase, including the trend of normalized velocity (i) and load in the y-direction (ii) under different *B*_1_ and *T*_3_ values. (H) Experimental results with the introduction of the glide phase, including the velocity trend under different *B*_1_ and *T*_3_ values (*n* = 3, data are presented as means ± SD) (i) and the robot’s profile during swimming at maximum velocity (ii). Scale bar: 5 mm.

To investigate the relationship between the specific parameters of the asymmetric trapezoidal magnetic field waveform and the motion state of the J-MSR, we constructed a testing platform (Fig. 2A) capable of real-time jellyfish position recording and developed a 2D multiphysics coupling numerical analysis method that integrates magnetic-fluid-solid mechanics (see the “Multiphysics coupling simulation of the J-MSRs” section in Methods). Prior to the experiments, we conducted simulation analyses and qualitative discussions (see Note S2) to streamline the exploratory experiments by adopting the following 3 simplifications: first, according to the limitations of the experimental setup, we fixed *T*_2_ = 0.01 s and *B*_2_ = −20 mT for analysis. Second, to ensure continuous swimming motion, we designed the waveform with equal slopes during *T*_4_ and *T*_1_ (*T*_4_ = *T*_1_ × *B*_2_/*B*_1_). Third, the variable *T*_3_ was analyzed independently to quantitatively evaluate its impact.

Based on these simplifications, experiments were conducted to investigate the effects of *B*_1_ and *T*_1_ on the swimming performance of J-MSRs. This experimental design specifically aimed to explore how variations in the preload phase influence overall performance, under the condition of keeping the parameters of the contraction phase unchanged. The values of *B*_1_ were set to 2.5, 5, 7.5, 10, 15, and 20 mT. The values of *T*_1_ started at 0.01 s and increased in increments of 0.01 s. When *T*_1_ ≥ 0.1 s, the increment was adjusted to 0.02 s per step, continuing until the J-MSRs were unable to complete the ascent motion. A total of 132 parameter combinations were tested, with each experimental group repeated 3 times to mitigate the impact of motion instability and measurement errors. The specific method for measuring and calculating the average velocity of J-MSR can be found in Note S3. The results, shown in Fig. 2B, indicate that as *T*_1_ increases, the speed of J-MSR decreases steadily and uniformly. The maximum speed for each value of the maximum positive magnetic flux density (*B*_1_) is almost always achieved when *T*_1_ is at its minimum value of 0.01 s. This suggests that *T*_1_ should be minimized when designing the waveform. Conversely, as *B*_1_ increases, the normalized swimming speed exhibits a trend of initially increasing and then decreasing. The peak swimming efficiency is achieved at *B*_1_ = 7.5 mT, and *T*_1_ = 0.01 s, where the J-MSR reaches its maximum speed of 13.02 body/s. Typical experimental results can be found in Movie S1.

To elucidate underlying causes of this phenomenon, we used simulation models to predict and analyze the entire swimming process of the J-MSR (Fig. 2C). During the preloading phase, the externally applied magnetic field is oriented upward, generating a magnetic torque ***τ***_1_ that induces a gradual upward bending of the J-MSR lappets. Subsequently, during the contraction phase, the magnetic field direction is reversed (*B*_2_ oriented downward), producing an oppositely directed magnetic torque ***τ***_2_ that drives a rapid downward bending motion of the lappets, resulting in effective propulsion. Further simulations were performed to evaluate the robot’s displacement over 5 cycles under varying *B*_1_ values when *T*_1_ = 0.01 s (Fig. 2D(i)). It is widely recognized that the robot does not reach a steady state in the initial cycles. Therefore, subsequent analyses focused on the last 3 cycles to evaluate propulsion performance. As shown in Fig. 2D(ii), the trend of average velocity changes in the simulations was consistent with experimental results, increasing initially and then decreasing as *B*_1_ increased, peaking at *B*_1_ = 7.5 mT. Previous studies have demonstrated that the area swept by the lappets is linearly proportional to the volume of fluid displaced [37], thereby serving as an indicator of the total force exerted by the fluid on the robot. For the J-MSR, the bending angle *θ*_1_ of the lappets is directly correlated with the swept area. As shown in Fig. 2E, the difference in lappet bending angle between its rise and fall stages diminishes as *B*_1_ increases. This reduction leads to a decreased disparity in the swept area between the preload and contraction phases, ultimately adversely affecting the swimming performance. This effect is also reflected in the y-direction fluid load exerted on the robot. When *B*_1_ = 5 and 7.5 mT, the peak fluid loads are comparable and higher than at *B*_1_ = 10 mT. This reduction in fluid load in *B*_1_ = 10 mT directly contributes to a decrease in the robot’s average speed. However, the longer recovery time required for *B*_1_ = 5 mT (purple region in Fig. 2E) also reduces the overall average speed during the cycle. Therefore, an appropriate *B*_1_ can increase the swept area of the J-MSR during the contraction phase, thereby enhancing the fluid load it experiences while maintaining a relatively short overall cycle time. At *B*_1_ = 7.5 mT, the nodal velocity and displacement of J-MSR in simulation are presented in Fig. 2F. The corresponding simulation animation can be found in Movie S2.

Further, we aim to introduce the glide phase (*T*_3_) into the simulation to investigate whether it can further enhance the propulsion performance of the robot, provide guidance for experiments, and ultimately reduce the number of experimental trials. As shown in Fig. 2G, we selected simulation results where *T*_3_ had a notable impact on the robot’s average speed (*B*_1_ = 5 to 10 mT). More details can be found in Movie S3. It was observed that the robot achieved the maximum average speed at *B*_1_ = 10 mT and *T*_3_ = 0.01 s, representing a 45.6% improvement compared to the condition without a glide phase. This finding demonstrates that an appropriate *T*_3_ allows the robot to maintain its contracted state longer, thereby increasing the fluid’s y-direction load on the robot (Fig. 2G(ii)). However, excessive *T*_3_ does not further enhance the fluid load; instead, it results in a reversing load during the recovery phase, diminishing propulsion efficiency. Based on the above analysis, we conducted experiments under identical magnetic field conditions (Fig. 2H). As depicted in Fig. 2H(i), the experimental results exhibit a trend consistent with the simulation, although a slight offset is observed in the conditions corresponding to the peak value. This offset might arise from asymmetric deformation of the J-MSR’s lappets during the experiment, particularly under larger *B*_1_ values, an effect not captured in the simulation. Even so, extending *T*_3_ enhances the robot’s stability, ultimately facilitating higher average speeds. Under the optimal waveform conditions (*B*_1_ = 10 mT, an appropriate *T*_3_), the robot achieved an impressive average speed of 14.85 BL/s. The corresponding motion trajectory is depicted in Fig. 2H(ii). More details can be found in Movie S4. It should be noted that the value of *B*_1_ corresponding to the optimal swimming performance of the J-MSR changed after the introduction of the glide phase (from 7.5 to 10 mT). This shift primarily occurs because the glide phase extends the duration of each motion cycle, thereby influencing the preload and contraction phases to some extent. Finally, we further evaluated the swimming performance of the J-MSR under the optimal actuation waveform in solutions with different dynamic viscosities. The results demonstrated that waveform optimization could still yield improvements even in high-viscosity fluids. Detailed results are provided in Note S4 and Movies S5 and S6.

Through the above mechanism analysis, we can delineate a relatively ideal jellyfish swimming process capable of rapid propulsion by optimizing its motion phases shown in Fig. 1D. The corresponding magnetic field and motion characteristics of each phase are described below. During the preload phase, an increasing magnetic field is applied, and then the J-MSR swings its lappets upward to store energy for the subsequent paddling motion. This phase can proceed quickly, but the final deformation (i.e., the magnetic field amplitude) should not be excessive. During the contraction phase, the lappets of the J-MSR rapidly transition from a slightly contracted upward position to a downward bell-like structure. At this stage, the magnetic field waveform should align with the robot’s morphological changes, rapidly decreasing from its maximum positive value to its maximum negative value. This phase is characterized by high speed and large shape deformation. The difference in lappet motion between the preload and contraction phases, such as the varying amplitude of shape changes, constitutes the spatial asymmetry and serves as the primary source of propulsion throughout the entire cycle. Subsequently, an appropriately designed glide phase is favorable to leverage the inertial effects of the robot’s kinematics. During this phase, the driving magnetic field remains at its maximum negative value to maintain the robot’s contracted state. This configuration allows the J-MSR to retain its bell-like structure, achieving an optimal hydrodynamic posture due to reduced fluid resistance compared to the fully expanded state. During the recovery phase, the J-MSR gradually transitions from the bell-like shape back to its natural relaxed state, preparing for the next cycle. The external magnetic field correspondingly decreases gradually from its maximum negative value to zero.

### Multimodal motion capabilities of the J-MSRs

**Fig. 3.**
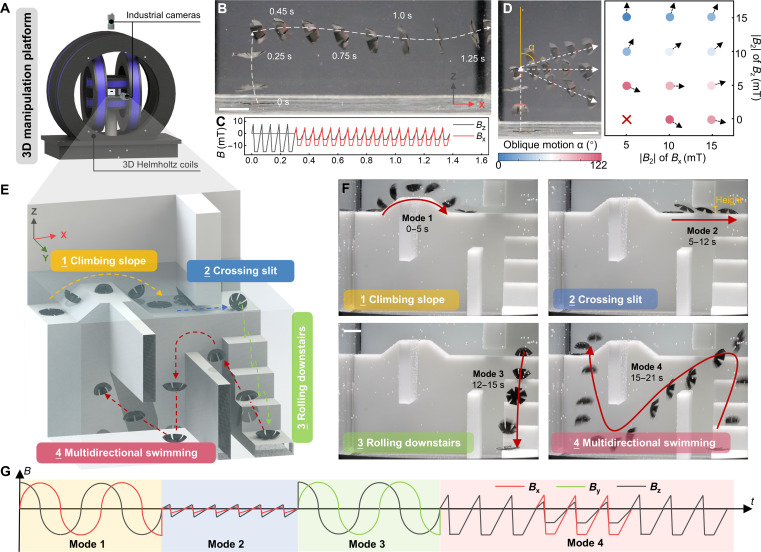
Demonstration of multimodal motion. (A) Schematic diagram of the developed 3D manipulation platform. (B) Swimming process of the jellyfish-inspired magnetic soft robot (J-MSR) at nearly horizontal angles. (C) Corresponding *B*_*x*_ and *B*_*z*_ magnetic field component diagrams during swimming. (D) Swimming direction control of the J-MSR under different *B*_*x*_ and *B*_*z*_ combinations, where the actuation waveform is uniformly scaled and quantified using the absolute value of |*B*_2_| within the waveform. (E) Environmental model for demonstrating the switching of multimodal motion. (F) Experimental motion decomposition diagrams of the J-MSR under different motion modes. (G) Schematic diagram of external magnetic control waveforms for different motion modes. Scale bar: 10 mm.

In clinical practice, micro magnetic robots enter the human body through natural orifices and perform various medical functions [38,39], offering higher safety and shorter postoperative recovery periods. However, performing medical tasks safely, reliably, and efficiently in such confined, complex environments requires these robots to not only possess excellent mobility but also have enough modalities to adapt to diverse environments [29,40]. The multimodal capabilities of magnetic robots depend on 2 key factors: their programmed internal magnetization, which is determined during the fabrication process, and the external driving magnetic field. To address this, we developed a 3D manipulation platform (Fig. 3A), which includes a 3-axis Helmholtz coil system (Fig. S5B, YP Magnetic Technology Development Co., Ltd., China) with dedicated power supplies, an industrial camera, and other components (more details are provided in Methods). This platform can generate a wider variety of external magnetic field driving modes. On this platform, we successfully demonstrated lateral swimming of J-MSR as shown in Fig. 3B, enabled by the application of bidirectional magnetic fields (Fig. 3C). It is noteworthy that both this experiment and subsequent tests utilized the optimal waveform identified in Fig. 2, along with its proportionally scaled variants. By adjusting the scaling ratio of the bidirectional magnetic fields, the J-MSR achieved large-angle deflection swimming ranging from 0° to 122° (Fig. 3D). Additional details can be found in Movie S7 and Note S5. Moreover, this actuation principle can be straightforwardly extended to the third orthogonal axis (***B***_*y*_), enabling full 3D orientation control in an analogous manner. To adapt to various fluidic environments within the body, we developed 3 distinct locomotion modes: rolling, crossing narrow slits, and multidirectional swimming. The rolling mode has been thoroughly validated in our previous studies [41,42], while information on the investigation of the crossing narrow slit mode can be found in Note S6. These modes were experimentally validated using a printed 3D model (Fig. 3E, fabricated by polylactic acid ). By tuning the driving magnetic field waveforms, we successfully demonstrated J-MSR performing tasks such as climbing slopes, crossing slits, rolling downstairs, and executing S-shaped swimming patterns, as illustrated in Fig. 3F and Movie S8. The waveforms used in each phase are also shown in Fig. 3G.

### Bioinspired functionalities of the J-MSRs

**Fig. 4.**
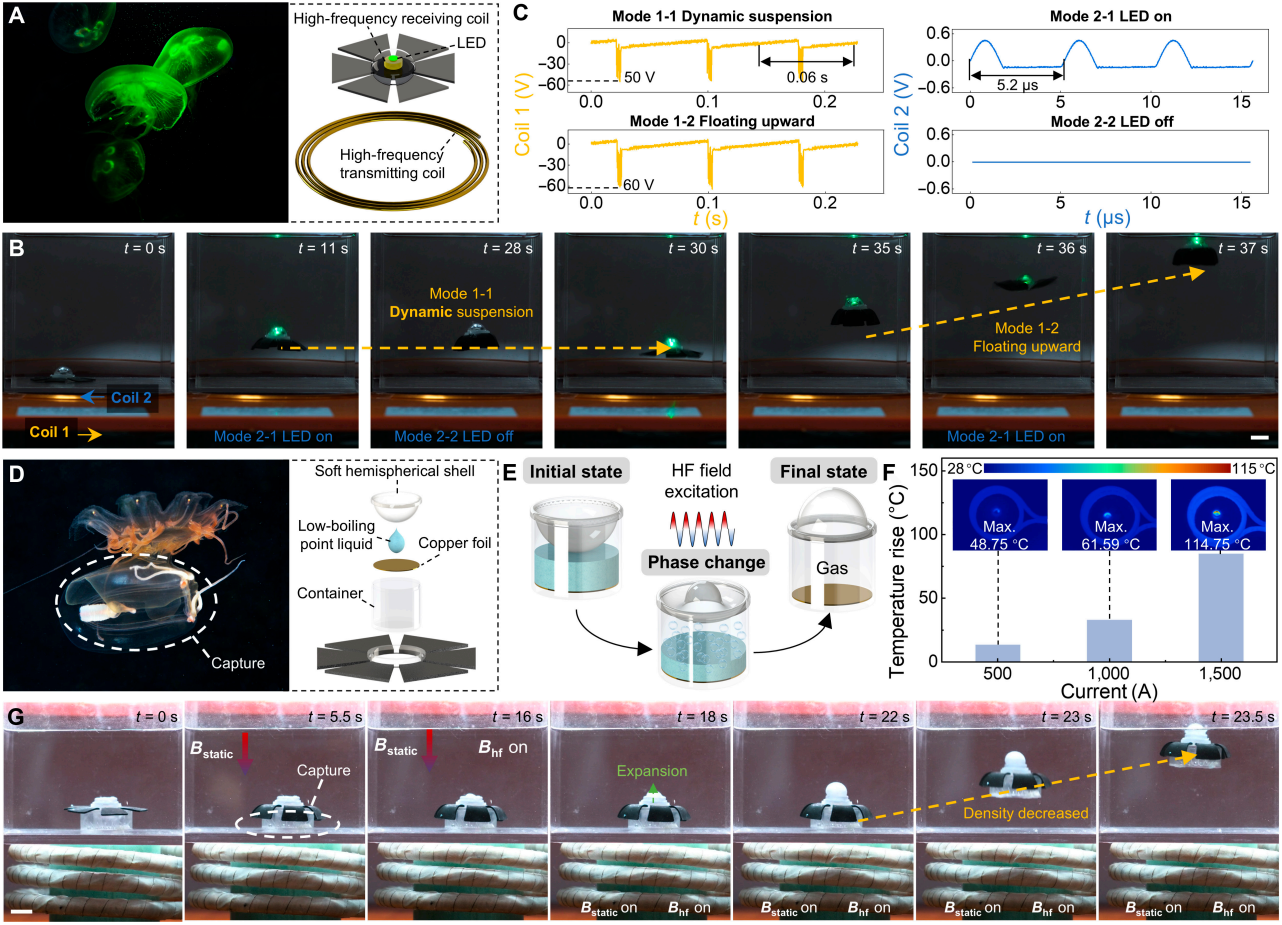
Bioinspired functions of the jellyfish-inspired magnetic soft robots (J-MSRs). (A) Robot design inspired by the bioluminescent behavior of jellyfish. (B) Experimental demonstration of robot swimming and illumination under dual-frequency magnetic actuation. (C) Voltage waveforms in Coil 1 and Coil 2 under different modes. The period of Coil 1 is 0.06 s (consistent with previous optimized results), with mode switching achieved through overall scaling. The period of Coil 2 is 5.2 μs, with mode switching achieved through switching. (D) Robot design inspired by the capture behavior of jellyfish, capable of performing capture actions while swimming. (E) Schematic diagram of the phase transition from liquid to gas in the variable-density device under high-frequency magnetic field (HF) driving. (F) Surface temperature rise of copper foils in the variable-density device under different input currents. (G) Experimental demonstration of robot’s capture behavior, where density decreases and the robot ascends after capturing an object under dual-frequency magnetic actuation. Scale bar: 10 mm. LED, light-emitting diode.

Jellyfish are renowned not only for their graceful swimming motions but also for their distinctive bioluminescence. As a critical ecological adaptation, bioluminescence is widely observed in marine organisms, including jellyfish [43], which aids in attracting prey. Inspired by this, we developed a scaled-up version of the original J-MSR (33.5 mm in diameter and 1.34 mm in thickness; Fig. S5). A 10-mm circular cavity was designed at its center to accommodate expandable functional modules, made possible by the robot’s excellent propulsion performance. By mimicking bioluminescent jellyfish, we integrated a light-emitting diode and a receiving coil at the central cavity of the J-MSR, with a high-frequency transmission coil (Fig. S8C) placed externally, as illustrated in Fig. 4A. As demonstrated in Fig. 4B, the J-MSR, equipped with a light emitting device, achieved dynamic suspension (Mode 1-1) and upward floating (Mode 1-2) under the control of external dual coils, while simultaneously flashing like a bioluminescent jellyfish (Movie S9). The corresponding voltage waveforms for Coil 1 and Coil 2 are shown in Fig. 4C. For Coil 1, the magnetic field waveform corresponds to the optimized magnetic actuation parameters identified in Fig. 2 (*B*_1_ = 10 mT, *T*_3_ = 0.02 s). By adjusting the amplification factor of Coil 1, the propulsion force generated by the robot can be modulated, enabling transitions between dynamic suspension and upward floating states. Meanwhile, the on-off state of the light-emitting diode is controlled via Coil 2 by switching its circuit, thereby toggling the J-MSR’s luminescence state.

Traditional jellyfish-like magnetically controlled robots face a fundamental limitation: They cannot simultaneously maintain swimming posture and execute grasping actions, which is the most reliable and efficient method for underwater object manipulation. To overcome this challenge, we integrated a variable-density device into the central cavity of the J-MSR. This device consists of a soft hemispherical shell, a low-boiling-point liquid, copper foil, and a container (Fig. 4D). The device’s initial state features a downward-concave soft hemispherical shell, with the liquid phase of the low-boiling-point fluid filling the container, as shown in Fig. 4E. When exposed to an external high-frequency magnetic field, the copper foil generates heat via electromagnetic induction, causing the liquid to vaporize. The increased internal pressure within the container drives the soft shell to deform upward. Ultimately, the soft hemispherical shell completely inverts into a convex shape, while the liquid inside fully transitions to gas, thereby reducing the overall density of the device. We first assessed the heating efficiency of the copper foil within the variable-density device under an induction heating machine (Fig. S8D, 45 kW, Guangdong Taiguan Power Technology Co., Ltd., China). As shown in Fig. 4F, after 15 s of heating with currents of 500, 1,000, and 1,500 A, the copper foil’s maximum temperature all surpassed the critical phase-transition point of the low-boiling-point liquid (>47 °C). To demonstrate the utility of this robot, we conducted underwater object-capture experiments (Fig. 4G). Initially, by applying a downward static magnetic field, the jellyfish robot successfully clamped onto the object (*t* = 5.5 s). Subsequently, a high-frequency magnetic field (38.5 kHz) was applied, triggering the phase transition within the variable-density device and resulting in noticeable expansion at *t* = 18 s. Finally, as the density of the device decreased below that of water, the J-MSR floated upward while securely gripping the object (Movie S10). Notably, the introduction of the high-frequency magnetic field, owing to its cycle being much shorter than the robot’s relaxation time, did not interfere with the robot’s swimming dynamics in the above 2 cases. More details can be found in Note S7 and Fig. S12. The synergistic interaction between the high frequency and the relatively low frequency required for the robot’s swimming greatly enhanced the J-MSR’s functionality, expanding its potential applications. Nevertheless, the present design is not intended for immediate in vivo use. For potential future in vivo applications, such as tissue interaction or object retrieval, materials and actuation strategies that strictly comply with physiological temperature and safety constraints would be required, including phase-change materials with body-compatible transition temperatures. In such cases, comprehensive thermal, mechanical, and biocompatibility analyses would be indispensable.

### Biomedical applications of the J-MSRs

**Fig. 5.**
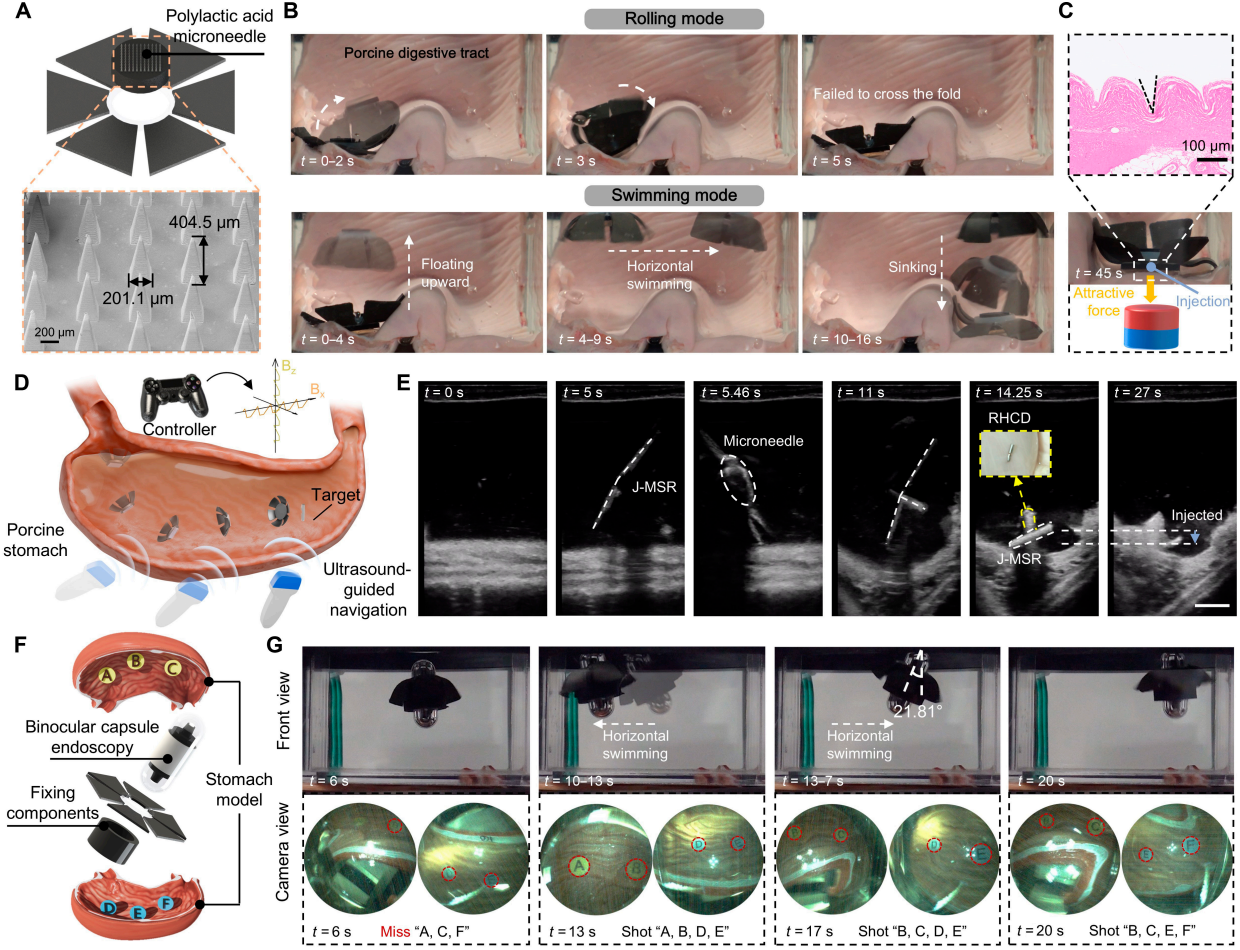
Biomedical applications of the jellyfish-inspired magnetic soft robots (J-MSRs). (A) Structural design of the robot equipped with microneedles, including scanning electron microscopy (SEM) images of the microneedles. (B) Experimental demonstration of the J-MSR’s different motion modes in an ex vivo pig stomach model. (C) After reaching the target area, the robot penetrates the tissue under the attraction of an external permanent magnet, including tissue section results. (D) Schematic diagram of the microneedle-equipped J-MSR navigating toward the target region inside an ex vivo porcine stomach under ultrasound guidance, with the target region indicated by the repositionable hemostasis clipping device (RHCD). (E) Experimental demonstration of the J-MSR under external magnetic actuation and ultrasound guidance, reaching the marker region and performing injection. (F) Schematic diagram of the robot equipped with a binocular capsule endoscopy and the functional test environment, with letters “A” to “F” representing different regions of the stomach. (G) Experimental demonstration of the J-MSR inspecting the stomach model under external magnetic actuation. Scale bar: 10 mm.

Magnetically controlled robots possess important advantages, such as noncontact operation, high controllability, and excellent penetration capability, making them promise to perform diverse medical tasks within bodily cavities and lumens (e.g., digestive systems). For example, as illustrated in Fig. 5A, by attaching a polylactic acid microneedle (designed by the authors and fabricated by Anhui Zhongdingyuxuan New Material Technology Co., Ltd.) to the central cavity of the J-MSR, the robot gains an injection capability, potentially enabling the delivery of macromolecular drugs. To validate this functionality, we constructed an ex vivo pig stomach model, simulating gastric folds commonly present in the stomach. As shown in Fig. 5B, the J-MSR initially attempted to cross the folds using a traditional rolling mode, similar to conventional robots, but failed. This further demonstrates the critical importance of multimodal transitions in unstructured environments such as the human body. By actuating the J-MSR to float upward, then swim horizontally, descend to a targeted region, and finally apply an external gradient force for anchoring, the robot successfully completed the injection. As demonstrated in Fig. 5C, the J-MSR effectively adapts to the complex, simulated in vivo environment, achieving precise targeted injection, with further details provided in Movie S11. To quantitatively assess its targeting performance, we first conducted benchtop experiments in a water tank under direct visual observation. Using a joystick-controlled 3D Helmholtz coil system, we generated specified actuation waveforms to guide the J-MSR toward predefined targets. A permanent magnet placed beneath the target provided additional attraction, enabling the microneedle-equipped J-MSR to leave a distinct imprint on the pressure-sensitive paper that marked the insertion site (Fig. S9A). Across 3 independent trials (*n* = 3, Fig. S9B), the targeting precision—defined as the Euclidean distance from the center of the premarked target (red cross) to the centroid of the J-MSR imprint—was (4.4 ± 1.85) mm. Relative to the 10 × 10 mm microneedle footprint, this corresponds to a normalized error of 0.44 ± 0.19 footprint widths.

Because direct visual guidance is not feasible in realistic biomedical procedures, we next performed an ultrasound-guided targeting experiment in an ex vivo porcine stomach model (Fig. 5D). A repositionable hemostasis clipping device was predeployed as an internal marker. The J-MSR was placed inside the ex vivo porcine stomach, which was filled with water and positioned at the center of a multiaxis square Helmholtz coil system (Fig. S10A). The square coil provided a sufficiently large workspace to allow stable placement of the ultrasound probe in close contact with the stomach surface (Fig. S10B). Under ultrasound guidance, the position of the J-MSR inside the stomach and the presence of the internal marker within the imaging field could be visualized. Based on it, the J-MSR was navigated toward the repositionable hemostasis clipping device and performed an injection adjacent to the marker (Fig. 5E), thereby demonstrating the feasibility of ultrasound-guided targeting in tissue. For more details, refer to Movie S12. After completing the first trial, the J-MSR was randomly repositioned to a distant location, and the procedure was repeated. In both trials, the J-MSR successfully reached the target site, yielding a 100% success rate. Notably, backward locomotion is readily achievable by simply reversing the direction of the applied magnetic field (equivalent to reversing the magnet orientation), as shown in Fig. S11.

The advent of magnetic capsule robot technology provides innovative solutions for previously challenging gastric examinations. However, traditional methods relying on static external magnetic fields to adjust capsule orientation often result in blind areas in the visual field [44]. The J-MSR addresses this limitation by incorporating a binocular capsule system (Fig. 5D) that enables simultaneous examination of both the upper and lower gastric regions. The design and system-level architecture of the custom-built binocular capsule endoscope, which consists of 2 GC0308 vision sensors, an ESP32-S3 microcontroller, and a power supply, are provided in Fig. S12. To evaluate the effectiveness of this functionality, we constructed a simulated gastric environment and affixed 6 letter markers (“A” to “F”) on the upper and lower halves of the stomach model to quantify the robot’s imaging coverage. As depicted in Fig. 5G, the J-MSR initially floated to a mid-level region, capturing only the “B”, “D”, and “E” markers (*t* = 6 s), which provided incomplete information. Subsequently, the robot was guided horizontally to the left by applying an external horizontal magnetic field, allowing it to capture “A”, “B”, “D”, and “E” (*t* = 13 s). Next, the horizontal field direction was reversed to move the robot to the right. During this process, the J-MSR achieved a maximum tilt angle of 21.81° (*t* =17 s), improving its ability to visualize blind spots in 3D surface. Finally, the robot reached the far-right side of the model, capturing “B”, “C”, “E”, and “F”, completing the simulated gastric examination. This innovative design greatly enhances the efficiency of endoscopic gastric inspections. For more details, refer to Movie S13.

## Discussion

In this work, we designed high-performance J-MSRs that integrate innovative, optimized magnetic field waveforms with a bioinspired magnetostructural design. Combining numerical simulations and experimental investigations, our study demonstrates that the optimized trapezoidal magnetic field waveform substantially enhances the locomotion performance of the J-MSR. The underlying enhancement mechanism lies in maximizing the spatial asymmetry resulting from the deformation difference between the preload and contraction phases, as well as leveraging inertial effects through the introduction of an appropriate glide phase, which collectively contribute to improved propulsion performance. These efforts enable the J-MSR to achieve ultrafast counter-gravity swimming at speeds up to 14.85 BL/s, even in environments with density differences exceeding 0.4 g/cm^3^. Notably, the robot operates without relying on buoyancy assistance and can switch seamlessly among multiple locomotion modes, including rolling, traversing narrow gaps, and multidirectional swimming. This design offers notable potential advantages for future medical applications, such as greater flexibility in density-based design, a compact structure ideal for confined spaces, improved stability through drift resistance, and highly adaptable movement. These advanced locomotion capabilities allow the J-MSR to perform complex tasks such as illumination, object capture, microneedle injection, and gastroscopy, demonstrating its broad potential for practical applications. The simultaneous integration of these functions within a single platform not only extends the capabilities of traditional single-function soft robots but also provides greater potential for future functional development. This multifunctional integration highlights the advantages of our magnetic actuation strategy and bioinspired locomotion design, enabling the J-MSR to demonstrate robust swimming performance and task versatility across diverse environments.

To further enhance the performance and translational potential of the J-MSRs, several directions merit exploration. First, although the proposed 2D-equivalent simulation offers computational efficiency and useful trend guidance, it cannot fully capture the robot’s real deformation, vortex dynamics, inter-lappet interactions, or the associated out-of-plane deformation. Future research could integrate 3D theoretical models such as discrete differential geometry [45] and Cosserat rod theory [46,47] to better clarify the complete motion dynamics of the J-MSRs from a fundamental mechanical perspective. Second, while this work demonstrates the considerable influence of multiple variables in the proposed trapezoidal waveform on the robot’s swimming performance, the performance achieved under a limited set of waveform parameters may not be globally optimal. Future work could explore the integration of machine learning-based approaches to enable the design and optimization of waveforms with higher degrees of freedom, or even arbitrary forms, thereby further enhancing the robot’s capabilities. In addition, the current control strategy primarily relies on open-loop control based on visual observation or ultrasound guidance, which may be limited in accuracy and robustness in complex and unstructured environments. Therefore, developing closed-loop autonomous control frameworks, together with quantitative accuracy metrics for navigation and actuation, remains an important direction for future research. Finally, although optimized actuation waveforms improve swimming performance under high-viscosity conditions, future work should evaluate J-MSR locomotion in biologically relevant mucus environments in vivo while systematically addressing practical challenges of coil-based magnetic actuation system, such as thermal management or the development of advanced hybrid magnetic actuation strategies [48], to further validate its biomedical and clinical potential.

## Data Availability

All data during this study are included in the text and the Supplementary Materials.

## Supplementary Materials

Supplementary 1 Notes S1 to S7 Figs. S1 to S19 Table S1 Movies S1 to S13

## References

[1] Ke X, Yong H, Xu F, Ding H, Wu Z. Stenus-inspired, swift, and agile untethered insect-scale soft propulsors. Nat Commun. 2024;15(1):1491. 38374180 10.1038/s41467-024-45997-3 PMC10876683

[2] Liu J, Li P, Huang Z, Liu H, Huang T. Earthworm-inspired multimodal pneumatic continuous soft robot enhanced by winding transmission. Cyborg Bionic Syst. 2025;6:0204. 40110346 10.34133/cbsystems.0204 PMC11919822

[3] Li G, Chen X, Zhou F, Liang Y, Xiao Y, Cao X, Zhang Z, Zhang M, Wu B, Yin S, et al. Self-powered soft robot in the Mariana Trench. Nature. 2021;591:66–71. 33658693 10.1038/s41586-020-03153-z

[4] Xie Z, Yuan F, Liu J, Tian L, Chen B, Fu Z, Mao S, Jin T, Wang Y, He X, et al. Octopus-inspired sensorized soft arm for environmental interaction. Sci Robot. 2023;8(84):eadh7852. 38019929 10.1126/scirobotics.adh7852

[5] Bang J, Choi SH, Pyun KR, Jung Y, Hong S, Kim D, Lee Y, Won D, Jeong S, Shin W, et al. Bioinspired electronics for intelligent soft robots. Nat Rev Electr Eng. 2024;1:597–613.

[6] Ye Z, Zheng L, Chen W, Wang B, Zhang L. Recent advances in bioinspired soft robots: Fabrication, actuation, tracking, and applications. Adv Mater Technol. 2024;9(21): Article 2301862.

[7] Coyle S, Majidi C, LeDuc P, Hsia KJ. Bio-inspired soft robotics: Material selection, actuation, and design. Extr Mech Lett. 2018;22:51–59.

[8] Costello JH, Colin SP, Dabiri JO, Gemmell BJ, Lucas KN, Sutherland KR. The hydrodynamics of jellyfish swimming. Annu Rev Mar Sci. 2021;13:375–396. 10.1146/annurev-marine-031120-091442 32600216

[9] Wang Y, Zhang P, Huang H, Zhu J. Bio-inspired transparent soft jellyfish robot. Soft Robot. 2023;10:590–600. 36577053 10.1089/soro.2022.0027

[10] Wang T, Joo H-J, Song S, Hu W, Keplinger C, Sitti M. A versatile jellyfish-like robotic platform for effective underwater propulsion and manipulation. Sci Adv. 2023;9(15): Article eadg0292. 37043565 10.1126/sciadv.adg0292 PMC10096580

[11] Cheng T, Li G, Liang Y, Zhang M, Liu B, Wong T-W, Forman J, Chen M, Wang G, Tao Y, et al. Untethered soft robotic jellyfish. Smart Mater Struct. 2018;28: Article 015019.

[12] Christianson C, Bayag C, Li G, Jadhav S, Giri A, Agba C, Li T, Tolley MT. Jellyfish-inspired soft robot driven by fluid electrode dielectric organic robotic actuators. Front Robot AI. 2019;6:126. 33501141 10.3389/frobt.2019.00126 PMC7806063

[13] Wang S, Chen Z. Modeling of two-dimensionally maneuverable jellyfish-inspired robot enabled by multiple soft actuators. IEEE/ASME Trans Mechatron. 2022;27(4):1998–2006.

[14] Wang S, Chen Z. Modeling of jellyfish-inspired robot enabled by dielectric elastomer. Int J Intell Robot Appl. 2021;5:287–299.

[15] Kazemi-Lari MAA, Dostine AD, Zhang J, Wineman AS, Shaw JA. Robotic jellyfish actuated with a shape memory alloy spring. Bioinspir Biomim Biorepl IX. 2019;10965:1096504.

[16] Villanueva A, Smith C, Priya S. A biomimetic robotic jellyfish (Robojelly) actuated by shape memory alloy composite actuators. Bioinspir Biomim. 2011;6: Article 036004. 21852714 10.1088/1748-3182/6/3/036004

[17] Yin C, Wei F, Fu S, Zhai Z, Ge Z, Yao L, Jiang M, Liu M. Visible light-driven jellyfish-like miniature swimming soft robot. ACS Appl Mater Interfaces. 2021;13(39):47147–47154. 34436851 10.1021/acsami.1c13975

[18] Ye J, Yao YC, Gao JY, Chen S, Zhang P, Sheng L, Liu J. LM-Jelly: Liquid metal enabled biomimetic robotic jellyfish. Soft Robot. 2022;9(6):1098–1107. 35486839 10.1089/soro.2021.0055

[19] Ren Z, Hu W, Dong X, Sitti M. Multi-functional soft-bodied jellyfish-like swimming. Nat Commun. 2019;10:2703. 31266939 10.1038/s41467-019-10549-7 PMC6606650

[20] Wang Q, Lu X, Yuan N, Jiang P, Yao J, Liu Y, Ding J. Centimeter-scale underwater robot with high-speed inspired by jellyfish. IEEE Robot Autom Let. 2023;8(5):2976–2982.

[21] Dai Y, Liang S, Chen Y, Feng Y, Chen D, Song B, Bai X, Zhang D, Feng L, Arai F. Untethered octopus-inspired millirobot actuated by regular tetrahedron arranged magnetic field. Adv Intell Syst. 2020;2: Article 1900148.

[22] Xu C, Yang Z, Lum GZ. Small-scale magnetic actuators with optimal six degrees-of-freedom. Adv Mater. 2021;33: Article 2100170. 10.1002/adma.202100170 33938046

[23] Ren Z, Wang T, Hu W, Sitti M. A magnetically-actuated untethered jellyfish-inspired soft milliswimmer. Paper presented at: Robotics: Science and Systems 2019. 2019 Jun 22–26; Freiburg im Breisgau, Germany.

[24] Qu J, Xu Y, Li Z, Yu Z, Mao B, Wang Y, Wang Z, Fan Q, Qian X, Zhang M, et al. Recent advances on underwater soft robots. Adv Intell Syst. 2024;6(2): Article 2300299.

[25] Zhou H, Mayorga-Martinez CC, Pané S, Zhang L, Pumera M. Magnetically driven micro and nanorobots. Chem Rev. 2021;121(8):4999–5041. 33787235 10.1021/acs.chemrev.0c01234 PMC8154323

[26] Peng Q, Huang J, Li C, Jiang M, Huang C, Luo J, Li H, Yin T, Cai M, Fu S, et al. Magnetically actuated soft electrodes for multisite bioelectrical monitoring of ex vivo tissues. Cyborg Bionic Syst. 2025;6:0434. 41142108 10.34133/cbsystems.0434 PMC12550281

[27] Xu Z, Chen Y, Xu Q. Spreadable magnetic soft robots with on-demand hardening. Research. 2023;6:0262. 38034084 10.34133/research.0262 PMC10687580

[28] Wen H, Shao Z, Sun Y, Ma C, Xiang F, Xia L, Zhu X, Li X, Li L, Cao Q. Magnetoactive bistable soft actuators for programmable large shape transformations at low magnetic fields. Nat Commun. 2025;16:9714. 41193499 10.1038/s41467-025-64855-4 PMC12589526

[29] Tian C, Mao L, Yang P, Zhang H, Meng X, Xie H. Carangiform-like magnetic milliswimmer with negative buoyancy for agile 3D navigation in confined fluid environments. Adv Funct Mater. 2024;35(1): Article 2411017.

[30] Pramanik R, Verstappen RWCP, Onck PR. Nature-inspired miniaturized magnetic soft robotic swimmers. Appl Phys Rev. 2024;11: Article 021312.

[31] Pramanik R, Verstappen RWCP, Onck PR. Magnetic-field-induced propulsion of jellyfish-inspired soft robotic swimmers. Phys Rev E. 2023;107: Article 014607. 36797941 10.1103/PhysRevE.107.014607

[32] Abrams MJ, Basinger T, Yuan W, Guo C-L, Goentoro L. Self-repairing symmetry in jellyfish through mechanically driven reorganization. Proc Natl Acad Sci USA. 2015;112(26):E3365–E3373. 26080418 10.1073/pnas.1502497112 PMC4491739

[33] Sapsis T, Peng J, Haller G. Instabilities on prey dynamics in jellyfish feeding. Bull Math Biol. 2011;73:1841–1856. 20976565 10.1007/s11538-010-9594-4

[34] Nawroth JC, Lee H, Feinberg AW, Ripplinger CM, McCain ML, Grosberg A, Dabiri JO, Parker KK. A tissue-engineered jellyfish with biomimetic propulsion. Nat Biotechnol. 2012;30:792–797. 22820316 10.1038/nbt.2269 PMC4026938

[35] Wu C, Xiang Y, Qu S, Song Y, Zheng Q. Numerical study of millimeter-scale magnetorheological elastomer robot for undulatory swimming. J Phys D Appl Phys. 2020;53: Article 235402.

[36] Zhao R, Kim Y, Chester SA, Sharma P, Zhao X. Mechanics of hard-magnetic soft materials. J Mech Phys Solids. 2019;124:244–263.

[37] Khaderi SN, Baltussen MGHM, Anderson PD, Ioan D, den Toonder JMJ, Onck PR. Nature-inspired microfluidic propulsion using magnetic actuation. Phys Rev E. 2009;79: Article 046304. 10.1103/PhysRevE.79.046304 19518330

[38] Nelson BJ, Pané S. Delivering drugs with microrobots. Science. 2023;382(6675):1120–1122. 38060660 10.1126/science.adh3073

[39] Wang C, Wu Y, Dong X, Armacki M, Sitti M. In situ sensing physiological properties of biological tissues using wireless miniature soft robots. Sci Adv. 2023;9: Article eadg3988. 37285426 10.1126/sciadv.adg3988 PMC7614673

[40] Li Q, Niu F, Yang H, Xu D, Dai J, Li J, Chen C, Sun L, Zhang L. Magnetically actuated soft microrobot with environmental Adaptative multimodal locomotion towards targeted delivery. Adv Sci. 2024;11: Article 2406600. 10.1002/advs.202406600 PMC11578324 39316063

[41] Sun Y, Zhang W, Gu J, Xia L, Cao Y, Zhu X, Wen H, Ouyang S, Liu R, Li J, et al. Magnetically driven capsules with multimodal response and multifunctionality for biomedical applications. Nat Commun. 2024;15:1839. 38424039 10.1038/s41467-024-46046-9 PMC10904804

[42] Sun Y, Ju Y, Wen H, Liu R, Cao Q, Li L. Hybrid-excited magneto-responsive soft actuators for grasping and manipulation of objects. Appl Mater Today. 2023;35: Article 101917.

[43] Widder EA. Bioluminescence in the ocean: Origins of biological, chemical, and ecological diversity. Science. 2010;328:704–708. 20448176 10.1126/science.1174269

[44] Song L, Dai Y, Wang L, Zhang W, Ji Y, Cao Y, Wei J, Wang F, Zhong J, Yang J, et al. Motion control of capsule robot based on adaptive magnetic levitation using electromagnetic coil. IEEE Trans Automat Sci Eng. 2023;20:2720–2731.

[45] Huang W, Huang X, Majidi C, Jawed MK. Dynamic simulation of articulated soft robots. Nat Commun. 2020;11:2233. 32376823 10.1038/s41467-020-15651-9 PMC7203284

[46] Renda F, Giorgio-Serchi F, Boyer F, Laschi C, Dias J, Seneviratne L. A unified multi-soft-body dynamic model for underwater soft robots. Int J Robot Res. 2018;37:648–666.

[47] Zhang X, Chan FK, Parthasarathy T, Gazzola M. Modeling and simulation of complex dynamic musculoskeletal architectures. Nat Commun. 2019;10:4825. 31645555 10.1038/s41467-019-12759-5 PMC6811595

[48] Sun Y, Zhu X, Li J, Chen S, Li L, Cao Q. Design and implementation of a hybrid magnetic actuation system for magnetic continuum intervention robots. Sensors Actuators A Phys. 2025;396: Article 117167.

