# Supplementary material for: Jellyfish-Inspired Ultrafast and Versatile Magnetic Soft Robots for Biomedical Applications
Source: Cyborg Bionic Syst. 2026 Apr 3;7:0540. doi: 10.34133/cbsystems.0540 (PMC13047275; doi:10.34133/cbsystems.0540)
Supplement: Supplementary 1 — Notes S1 to S7 Figs. S1 to S19 Table S1 Movies S1 to S13 [file cbsystems.0540.f1.zip › Supplementary material20260213.docx]

Supplementary Materials

**Jellyfish-Inspired Ultrafast and Versatile Magnetic Soft Robots for Biomedical Applications**

Yuxuan Sun^1,2,3^, Ruiqi Liu^1,2^, Chiyuan Ma^1,2^, Jingyang Liu^1,2^, Semina Yi^3^, Junnan Gu^4^, Liangyu Xia^1,2^, Haitao Qing^3^, Kailin Cai^5^, Liang Li^1,2^, Lining Yao^3*^, Quanliang Cao^1,2*^

^1^Wuhan National High Magnetic Field Center, Huazhong University of Science and Technology, Wuhan, 430074, China.

^2^School of Electrical and Electronic Engineering, Huazhong University of Science and Technology, Wuhan, 430074, China.

^2^State Key Laboratory of Advanced Electromagnetic Technology, Huazhong University of Science and Technology, Wuhan, 430074, China.

^3^Department of Mechanical Engineering, University of California, Berkeley, Berkeley, 94704, USA

^4^Department of Thoracic Surgery, Union Hospital, Tongji Medical College, Huazhong University of Science and Technology, Wuhan 430022, China.

^5^Department of Gastrointestinal Surgery, Union Hospital, Tongji Medical College, Huazhong University of Science and Technology, Wuhan, 430022, China.

*Corresponding authors. Email: liningy@berkeley.edu (L. Yao), quanliangcao@hust.edu.cn (Q. Cao.)

**The PDF file includes:**

Note S1. Discussion on the Geometric Parameters of the J-MSR’s Lappets

Note S2. Simplifications and Assumptions Prior to Experiments.

Note S3. Measurement and Calculation Process of J-MSR's Average Speed.

Note S4. Influence of fluid viscosity on J-MSR locomotion.

Note S5. Exploration and Analysis of Multidirectional Motion.

Note S6. Exploration and Analysis of Crossing Slit Motion.

Note S7. Dynamic Analysis of J-MSR under Superimposed High- and Low-Frequency Magnetic Fields

Fig. S1. Bio-inspired Design Process of the J-MSRs.

Fig. S2. Fabrication Process of the J-MSRs.

Fig. S3. Schematic illustration of the magnetization system for the J-MSR.

Fig. S4. Mechanical testing and material properties of the J-MSR.

Fig. S5. Dimension diagram of the J-MSR.

Fig. S6. Schematic diagrams of 1D test platform and 3D manipulation platform.

Fig. S7. Finite element mesh of the J-MSR in simulations.

Fig. S8. Pictures of magnetic field generating devices.

Fig. S9. Targeting performance of J-MSR in a water tank under direct visual observation.

Fig. S10. Experimental setup for ultrasound-guided targeting in an ex vivo porcine stomach.

Fig. S11. Reversible locomotion of the J-MSR enabled by magnetic field reversal.

Fig. S12. Design and system-level architecture of the binocular capsule endoscope integrated with the J-MSR.

Fig. S13. Schematic of the thrust analysis equivalent model.

Fig. S14. Simulation study on the settings of T2 and B2.

Fig. S15. Displacement curve and images of the J-MSR’s motion process at B1=5mT and T1 =0.03s.

Fig. S16. Effect of fluid viscosity on the swimming performance of the J-MSR.

Fig. S17. Improvement of J-MSR locomotion in high-viscosity fluids (50 mPa·s) by optimizing the actuation waveform.

Fig. S18. Images of the J-MSR's motion process as it crosses through slits of different heights.

Fig. S19. Dynamic response of the J-MSR under superimposed low- and high-frequency magnetic fields.

Table S1. Comparison of the proposed jellyfish-inspired robots with the ones previously reported in the literature.

**Other Supplementary Material for this manuscript includes the** **following.**

Video S1 (.mp4 format). Swimming process without glide phase under different B_1_ (Experiment).

Video S2 (.mp4 format). Swimming process without glide phase under different B_1_ (Simulation).

Video S3 (.mp4 format). Swimming process with glide phase under different T_3_ (Simulation).

Video S4 (.mp4 format). Swimming process with glide phase under different T_3_ (Experiment).

Video S5 (.mp4 format). Swimming performance in fluids with different viscosities

Video S6 (.mp4 format). Swimming performance in high-viscosity fluid under different B_1_

Video S7 (.mp4 format). Oblique swimming.

Video S8 (.mp4 format). Multimodal motion.

Video S9 (.mp4 format). Luminescence.

Video S10 (.mp4 format). Density Change.

Video S11 (.mp4 format). Biomedical application: Injection (under direct visual observation).

Video S12 (.mp4 format). Biomedical application: Injection (under ultrasound guidance).

Video S13 (.mp4 format). Biomedical Application: Gastroscopy.

**Supplementary Notes:**

**Note S1. Discussion on the Geometric Parameters of the J-MSR’s Lappets**

To assess how lappet’s geometry influences the propulsion performance of the jellyfish-inspired magnetic soft robot (J-MSR), we adopted the thrust‐equivalent model ^[1]^ and analyzed the effects of key geometric ratios—specifically the lappet width ratio and the inner‐to‐outer radius ratio—on thrust generation to guide the geometric optimization of the J‑MSR. Each lappet is represented by an n‑layer, radially arranged, continuous array of cylinders (Fig. S13a), where *r_1_*, *r_2_*, …, *r_n_* denote the characteristic radial positions of layers 1 through *n*. When “unwrapped” into a linear array (Fig. S13b), each layer *i* comprises cylinders of diameter *b_i_* separated by gaps of width *a_i_*. Accordingly, the local porosity *P_i_* of layer *i* can be expressed as:

|  | $P_{i}=\frac{a_{i}}{a_{i}+b_{i}}\times100\%$ | (S1) |
| --- | --- | --- |

The local fluid velocity *U_i_* at the *i*‑th layer of the cylindrical array is given by

|  | $U_{i}=r_{i}\times\omega$ | (S2) |
| --- | --- | --- |

Where *ω* is the angular velocity of the fluid.

Taking *ν* the kinematic viscosity and using the Reynolds number *R* to characterize the flow regime, the local Reynolds number *R_i_* at the *i*‑th layer of the cylindrical array can be calculated as:

|  | $R_{i}=\frac{U_{i}b_{i}}{\upsilon}$ | (S3) |
| --- | --- | --- |

The local drag coefficient of the *i*‑th layer of the cylindrical array can be expressed as a function of the local porosity *P_i_* and the Reynolds number *R_i_* [2]. The specific expression is given by:

|  | $C_{i}^{D}=f\left(P_{i},R_{i}\right)$ | (S4) |
| --- | --- | --- |
|  | $x_{i}=\frac{2\left(1+\Delta_{i}^{4}\right)}{2\left(1+\Delta_{i}^{4}\right)+\ln\left(\Delta_{i}\right)-\Delta_{i}^{4}+1}$ | (S5) |
|  | $\Delta_{i}=\frac{1}{\sqrt{1-P_{i}}}$ | (S6) |

To determine the total hydrodynamic thrust *F_dr_* acting on the J-MSR with a specific lappet geometry, the local drag coefficients of each cylindrical layer must be summed. Accordingly, the total thrust can be expressed as:

|  | $F_{dr}=\sum_{i=1}^{n} \frac{1}{2}\rho C_{i}^{D}A_{i}U_{i}^{2}$ | (S7) |
| --- | --- | --- |

In this equation, to obtain a sufficiently accurate thrust estimation, the number of cylindrical layers *n* should be as large as possible to enhance the approximation accuracy of the integral. Here, *A_i_* denotes the local reference area of the *i*-th cylindrical array, which corresponds to the projected area facing the incoming flow and is given by:

|  | $A_{i}=Nb_{i}\left(r_{i+1}-r_{i}\right)$ | (S8) |
| --- | --- | --- |

Where *N* represents the number of J-MSR’s lappets.

The primary design consideration for the geometry of the J-MSR lies in the dimensional configuration of its lappets. The lappet geometry is characterized by four key parameters: inner diameter *l_i_*, outer diameter *l_o_*, inner width *w_i_*, and outer width *w_o_*, as illustrated in Fig. S13c.

To further describe the geometric configuration, the lappet diameter ratio l_γ_ and width ratio are defined as:

|  | $l_{\gamma}=\frac{l_{i}}{l_{o}}$ | (S9) |
| --- | --- | --- |
|  | $w_{\gamma}=\frac{w_{o}}{w_{i}}$ | (S10) |

By substituting the inner-to-outer diameter ratio *l_γ_* and the lappet width ratio *w_γ_* of the J-MSR into Equation (S7), the influence of geometric parameters on the total propulsive force can be analyzed. A larger *w_γ_* theoretically yields a greater thrust; however, an excessively large *w_γ_* may induce undesirable hydrodynamic interactions between adjacent lappets. Based on the parameters reported in references [1,2], the inner-to-outer diameter ratio is set to 1:1.8 to achieve optimal actuation performance, and the inner-to-outer lappet width ratio is selected as 0.44:1.

**Note S2. Simplifications and Assumptions Prior to Experiments.**

In practice, we found that simultaneously investigating the effects of all six parameters on the J-MSR’s motion state significantly complicates the analysis. Each parameter has a specific range of values, and exploring all possible combinations would result in a geometrically increasing number of experiments. Furthermore, the effects of these parameters are interdependent, making it challenging to independently analyze the influence of each parameter on the motion state. To address this complexity, we streamlined the exploratory experiments by assigning and consolidating the waveform parameters based on the optimization objectives and the inherent motion process of the J-MSR. This approach includes the following three key aspects.

First, during the contraction phase, the lappet's paddling motion must achieve sufficient speed to generate enough kinetic energy from the reactive force of the surrounding liquid to counteract the downward displacement caused by the difference between gravity and buoyancy throughout the motion cycle. To validate this, we designed a specific magnetic waveform with only contraction phase (Fig. S14a) and calculated the average swimming speed of the J-MSR from the initial to the final state (Fig. S14b). The distribution of average speeds under various combinations of contraction duration (T_2_) and reverse peak magnetic induction amplitude (B_2_) is shown in Fig. S14c. It can be observed that a shorter contraction duration and a larger reverse magnetic field amplitude led to enhanced propulsion performance. The developed 1D test platform can generate a maximum unidirectional uniform magnetic field of 20 mT in space. Accordingly, B_2_ is set to 20 mT. Considering the limitations of the power amplifier’s feedback loop adjustment capabilities and the physical constraint that current cannot change abruptly under inductive loads, the transition time of the driving current from the positive maximum to the negative maximum should not be too short (>10 ms). Therefore, T_2_ is set to 0.01 s. The above simplification also facilitates the quantification of the spatial asymmetry effect in the J-MSR's swimming process.

Second, to ensure continuous swimming motion, the driving waveform is designed to maintain the same slope during T_4_ and T_1_ (T_4_=T_1_*B_2_/B_1_), thereby transforming T_4_ from an independent variable to a dependent variable. This constraint preserves waveform continuity, ensuring a smooth connection between the end of T_4_ and the beginning of the next cycle, with consistent slopes before and after the transition. Such a design allows the robot to move gently without abrupt jerks, enhancing its motion stability. Simultaneously, it reduces the complexity of parameter settings, facilitating smoother experimental implementation.

Finally, the duration of the glide phase (T_3_) can be isolated from other parameters for independent analysis. During this phase, the driving waveform remains constant, meaning that the J-MSR maintains its current state without altering its posture, relying solely on inertia for movement. At this stage, the robot is influenced only by gravity and buoyancy, resulting in a relatively simple and predictable motion state. Moreover, parameter T_3_ solely determines the duration of the robot’s inertial gliding and exhibits minimal coupling with other parameters. Therefore, it is analyzed independently.

**Note S3. Measurement and Calculation Process of J-MSR's Average Speed.**

The experimental setup consisted of a vertical motion space approximately 150 mm in height. To ensure reliable measurements, the analysis excluded the bottom and top sections of the displacement curve. Near the bottom, the complex fluid environment and reactive forces from the container walls could distort the true motion capabilities of the robot. Similarly, near the top, the liquid-air interface and surface tension effects introduced uncontrollable motion. Thus, the middle section of the displacement curve, corresponding to heights between 30 mm and 100 mm from the bottom, was used to calculate the average swimming speed. Specific calculation points were selected from the mid-portion of a displacement cycle, where the slope was parallel to the tangent of the curve's overall trajectory. The swimming efficiency of the J-MSR was evaluated using normalized swimming speed, defined as the ratio of velocity to body length.

Using the parameters B_1_=5mT and T_1_ =0.03s as an example, the specific measurement and calculation process is described as follows. Given T_3_=0 s, T_4_ =0.12s is calculated using Equation (1), thereby determining all parameters of the driving waveform. These parameters are then input into the host computer, which calculates the required driving waveform for the experiment through the microcontroller. After pressing the start button, a magnetic field in the Z-axis direction is generated. The entire motion process of the J-MSR is recorded using an industrial camera. When the robot reaches the top of the liquid surface, the driving waveform output is stopped, and recording ends. The experiment is repeated three times under the same parameters and experimental conditions, producing three sets of displacement curves, as shown in Fig. S15. The bottom right corner of the figure displays the video frame count, with each frame corresponding to 1/240 s. The average speed of J-MSR under the given magnetic field waveform is obtained by calculating the average of the slopes of the displacement curves from the three trials within a specified interval.

**Note S4. Influence of fluid viscosity on J-MSR locomotion**

Since fluid viscosity plays a critical role in locomotion, we further examined the swimming performance of the J-MSR in fluids with dynamic viscosities ranging from 5 to 50 mPa·s (n ≥ 3 per condition). Test fluids were prepared to target the desired dynamic viscosities and verified on a rotational rheometer prior to each session. For each condition, the optimal actuation waveform (B_1_= 7.5 mT, T_1_= 0.01 s, B_2_=-20 mT, T_2_=0.01 s, T_3_= 0.02 s, T_4_= 0.0267 s) was consistently applied. Motion trajectories were captured and analyzed using a frame-by-frame tracking software (See "Methods" section) to extract instantaneous positions, and the reported swimming speed was calculated as the mean value over steady-state in each trial.

The results show a monotonic and steep decline in swimming speed with increasing viscosity. Notably, the J-MSR was nearly unable to sustain upward swimming at 50 mPa·s as shown in Fig. S16a and Video. S5. Kinematically, the increased dynamic viscosity exerted markedly greater resistance on the lappets of J-MSR, which in turn led to a reduction in the maximum upward deflection angle during the energy-storage phase from 114.2° to 65.7° with increasing viscosity. This significantly reduced the effective swept area of the lappets within a single cycle of the J-MSR and, consequently, diminished thrust generation. In addition, the experimental results exhibited good consistency with the trends predicted by the constructed simulation model, as shown in Fig. S16b.

In the experimental observations, it was evident that at higher viscosities the J-MSR struggled to achieve effective upward strokes. To address this limitation, we increased the value of B₁ and found that swimming performance could still be improved even in 50 mPa·s fluid (Fig. S17b and Video. S6). Specifically, the maximum upward deflection angle during the energy-storage stage increased from 65.7° to 90°, and similar improvements were also confirmed in the simulations (Fig. S17a). This finding demonstrates that, under higher viscosity conditions, optimizing the actuation waveform becomes critical for enabling effective locomotion. Standard deviations of swimming speed across repeated trials have now been included in the added figure to demonstrate reproducibility.

**Note S5. Exploration and Analysis of Multidirectional Motion.**

Take |B_2_| of B_z_ =5 mT, |B_2_| of B_x_ = 10 mT as an example, the specific motion process of J-MSR under this waveform combination is shown in Fig. 3b. In the initial phase, driven by the unidirectional magnetic field along the Z-axis, the robot ascends rapidly in the vertical direction. After approximately 5 motion cycles (about 0.3 s), the robot reaches a height of about 25 mm from the bottom of the container, exiting the area where it relies on reactive forces generated by the fluid along the container wall. Subsequently, the robot is simultaneously influenced by the Z-axis and X-axis driving magnetic fields. The robot begins to tilt and swims with an inclined posture of approximately 70°, ultimately reaching the right wall of the container at 1.375 s, completing the motion process. By measuring the position where the inclined motion begins and the point of contact with the right wall, the robot's inclined motion angle in this experiment is calculated to be 91.1^°^.

A total of 12 groups of experiments were conducted with different parameter combinations, and the results are shown in Fig. 3d. The black arrows indicate the actual motion directions of J-MSR in space under the corresponding magnetic field conditions. With the increase of |B_2_| of B_z_, J-MSR tends to move in the vertical direction. Conversely, as |B_2_| of B_x_ increases, the robot tends to move in the horizontal direction. The Z-axis driving magnetic field primarily provides the upward force to counteract the robot's weight, enabling upward swimming. The X-axis magnetic field serves both a guiding and driving function, tilting the robot's posture and supplying the propulsion for horizontal motion. When the Z-axis driving magnetic field is weak, the robot exhibits an inclination angle greater than 90^∘^, moving toward the lower-right direction. This occurs because the Z-axis magnetic field is the robot's main source of upward propulsion. With a lower amplitude, the driving force derived from the external field is insufficient to overcome the combined effects of gravity and buoyancy, resulting in a smaller net vertical force. Meanwhile, the horizontal magnetic driving force remains strong and dominates, causing the robot to move diagonally downward.

**Note S6. Exploration and Analysis of Crossing Slit Motion.**

In the J-MSR’s slit traversal experiment, the limited height of the slits inhibits the formation of a complete bell-shaped structure, distinguishing this scenario from conventional swimming environments. Therefore, rather than employing the traditional jellyfish-like deformation propulsion, the robot depends on high-frequency, minimal deformations to move. Achieving this deformation necessitates a significant reduction in the magnetic induction intensity amplitude of the driving waveform. The slit heights were determined based on the measured height of the bell-shaped structure formed by the jellyfish-inspired magnetic soft robot during downward contraction. The structure's height was measured as h=3.4  mm, leading to the selection of three slit heights: 0.5*h, 1.0*h, 1.5*h. For the driving waveform, a short-period configuration was employed, utilizing unidirectional drive without a glide phase to enhance performance. The time parameters were set as T_1_=0.01 s，T_2_=0.01 s，T_3_=0 s，T_4_=0.027 s. The magnetic induction intensity B_1_ was derived from Equation (S1), and B_2_ was scaled to correspond to the different slit heights.

|  | $B_{1}=\frac{7.5}{20}\times B_{2}$ | (S1) |
| --- | --- | --- |

The robot's traversal through slits of varying heights is depicted in Fig. S18, with the associated magnetic field parameters shown in the top-right corner. Driven by a low-amplitude magnetic field, the J-MSR employs high-frequency flapping motions to crawl along the surface and pass through narrow slits. Remarkably, it can pass through slits as low as 0.5 times its bell-shaped structure height.

**Note S7. Dynamic Analysis of J-MSR under Superimposed High- and Low-Frequency Magnetic Fields**

To assess whether the introduction of a high-frequency magnetic field alters the dynamic behavior of the J-MSR, we superimposed a 10 kHz sinusoidal field onto the original background magnetic field (Fig. S19a). Three high-frequency field amplitudes (0 mT, 0.1 mT, and 1 mT) were tested, while the background field parameters were held constant at T₁ = 0.01 s, T₂ = 0.01 s, T₃ = 0 s, T₄ = 0.02667 s, B₁ = 7.5 mT, and B₂ = –20 mT. Focusing on the contraction phase, point probes were placed at the J-MSR’s geometric center and at the tip of a lappet to record their displacements over time (Fig. S19b). The resulting displacement traces demonstrate that, because the high-frequency field’s cycle is orders of magnitude shorter than the robot’s relaxation time, it does not perturb the swimming dynamics. Furthermore, in Fig. 4 of the main text, the applied high-frequency fields (38.5 kHz and 192 kHz) are well above the frequency considered here, and thus likewise exert no observable influence on the J-MSR’s motion.

**Supplementary Figures:**

**
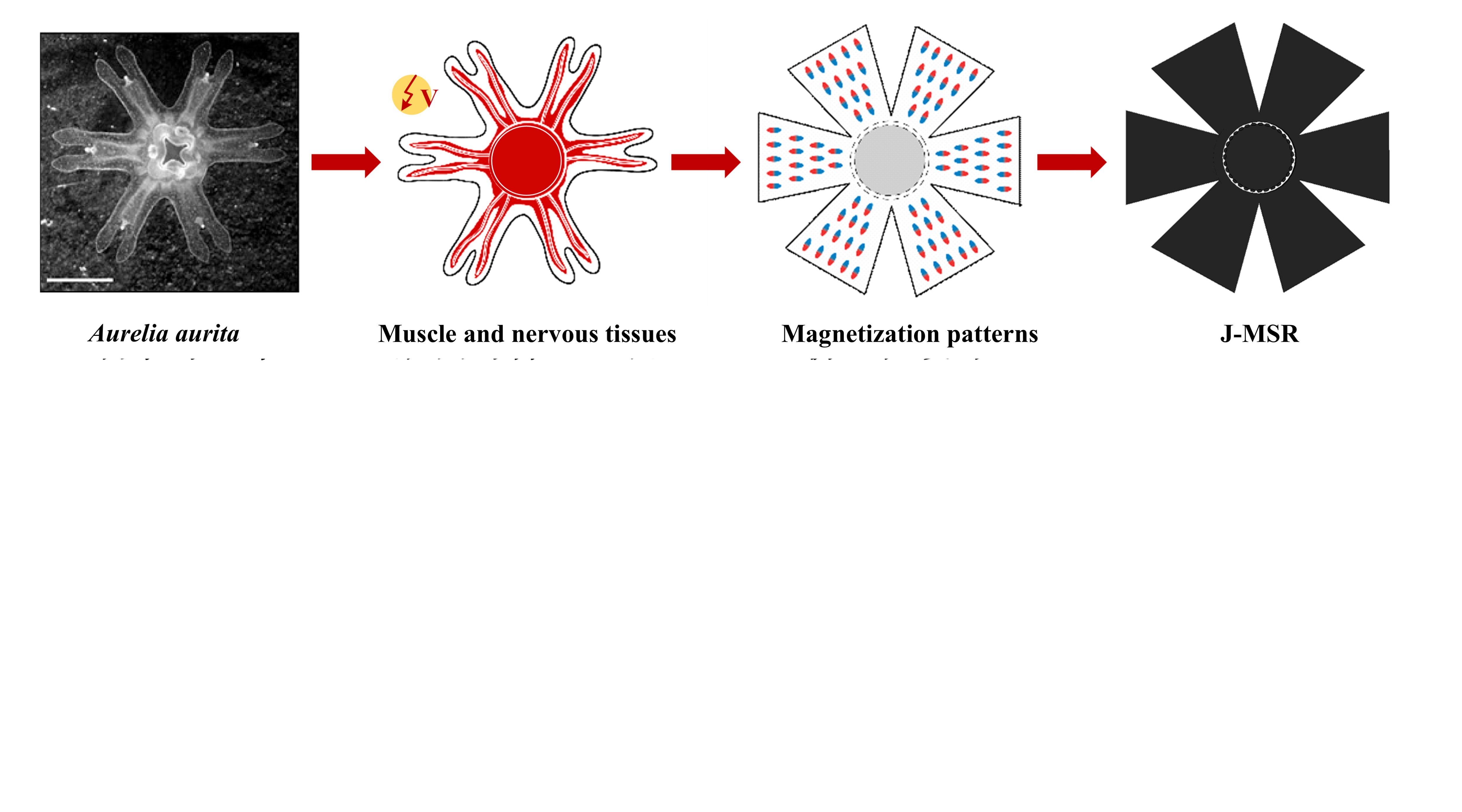
**

**Fig. S1. Bio-inspired Design Process of the J-MSRs**.


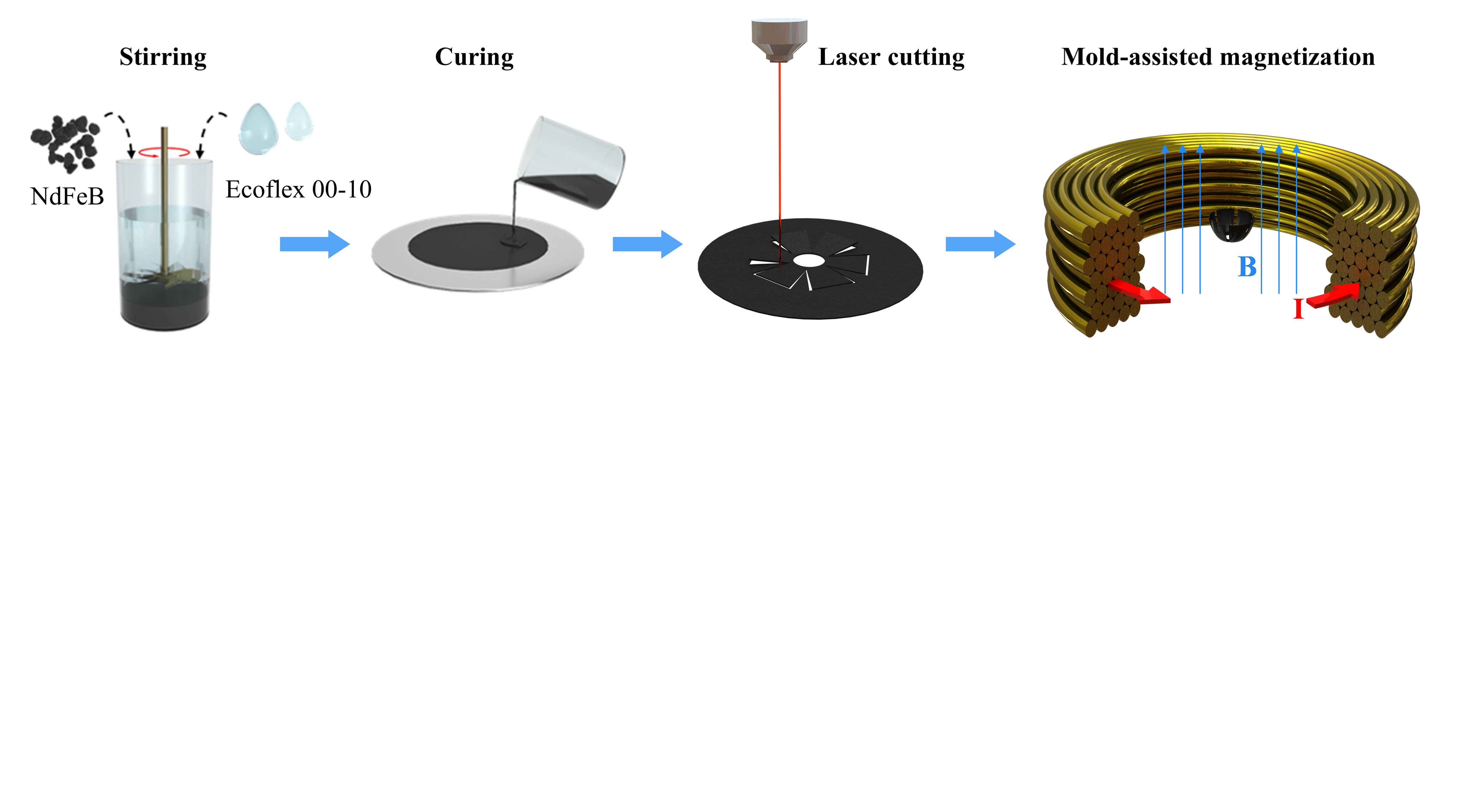


**Fig. S2. Fabrication Process of the J-MSRs**.


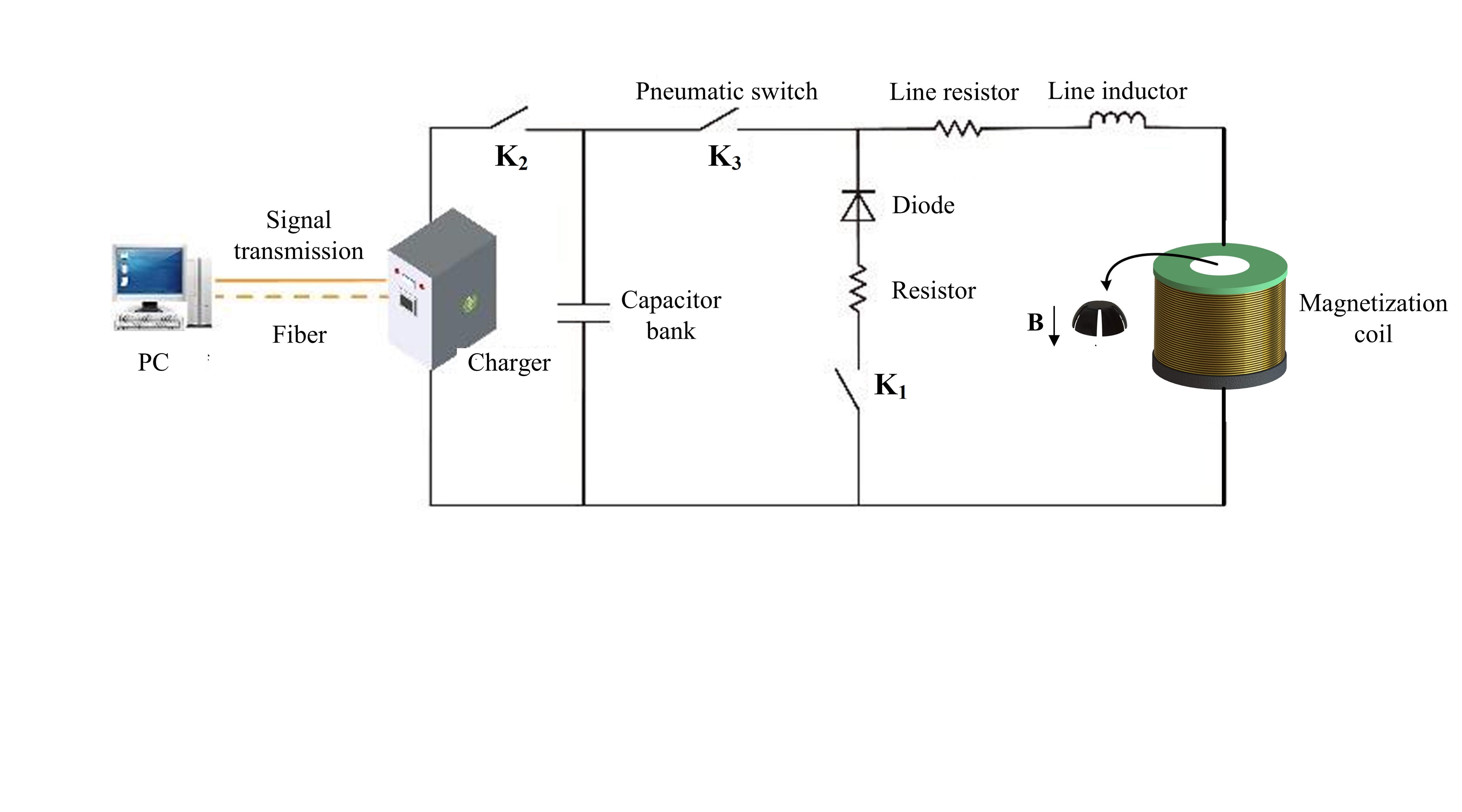


**Fig. S3. Schematic illustration of the magnetization system for the J-MSR.**

*
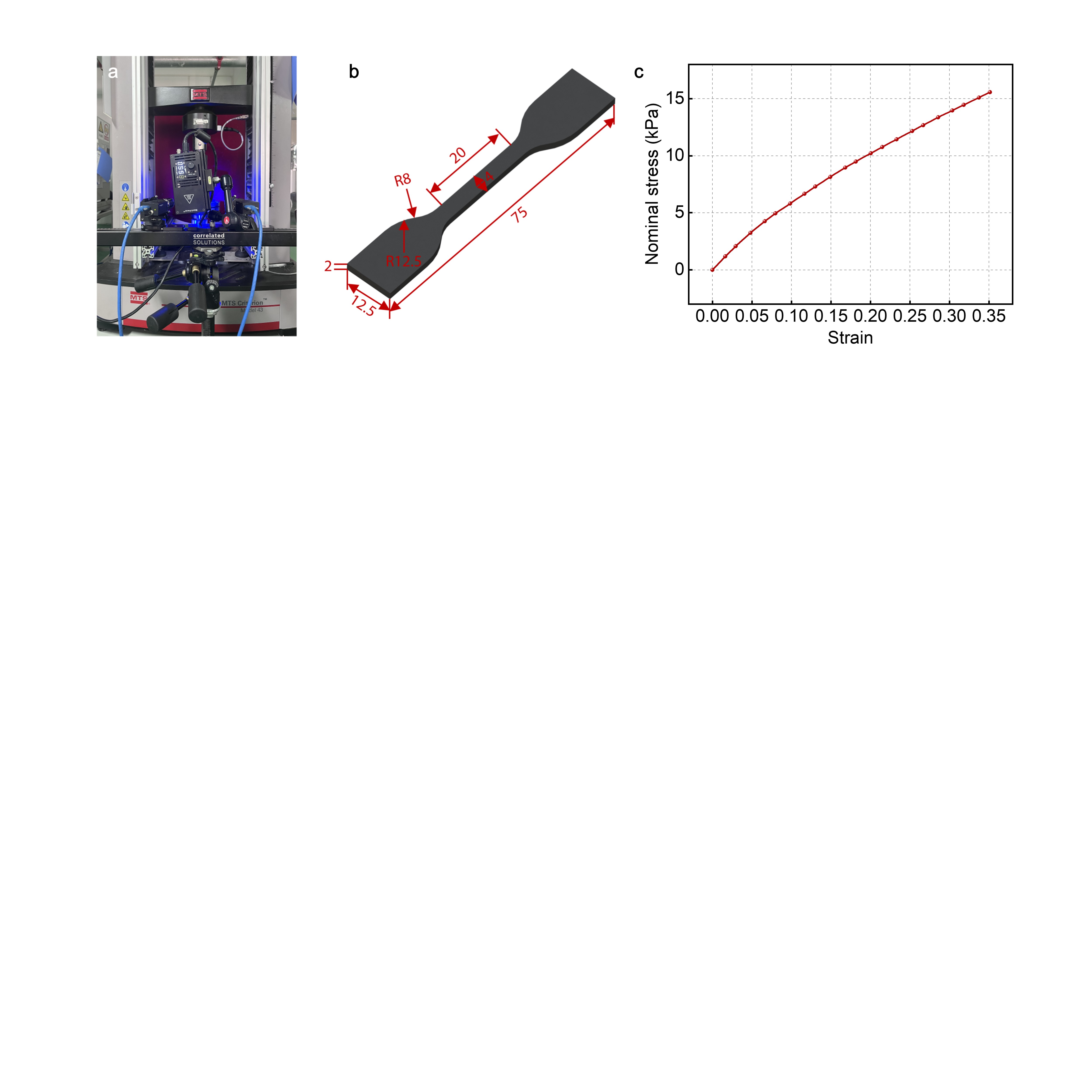
*

**Fig. S4. Mechanical testing and material properties of the J-MSR.** (a) Experimental setup for uniaxial tensile testing. (b) Geometric dimensions of the specimen. (c)Nominal stress-tension curves. All units are in millimeters**.**


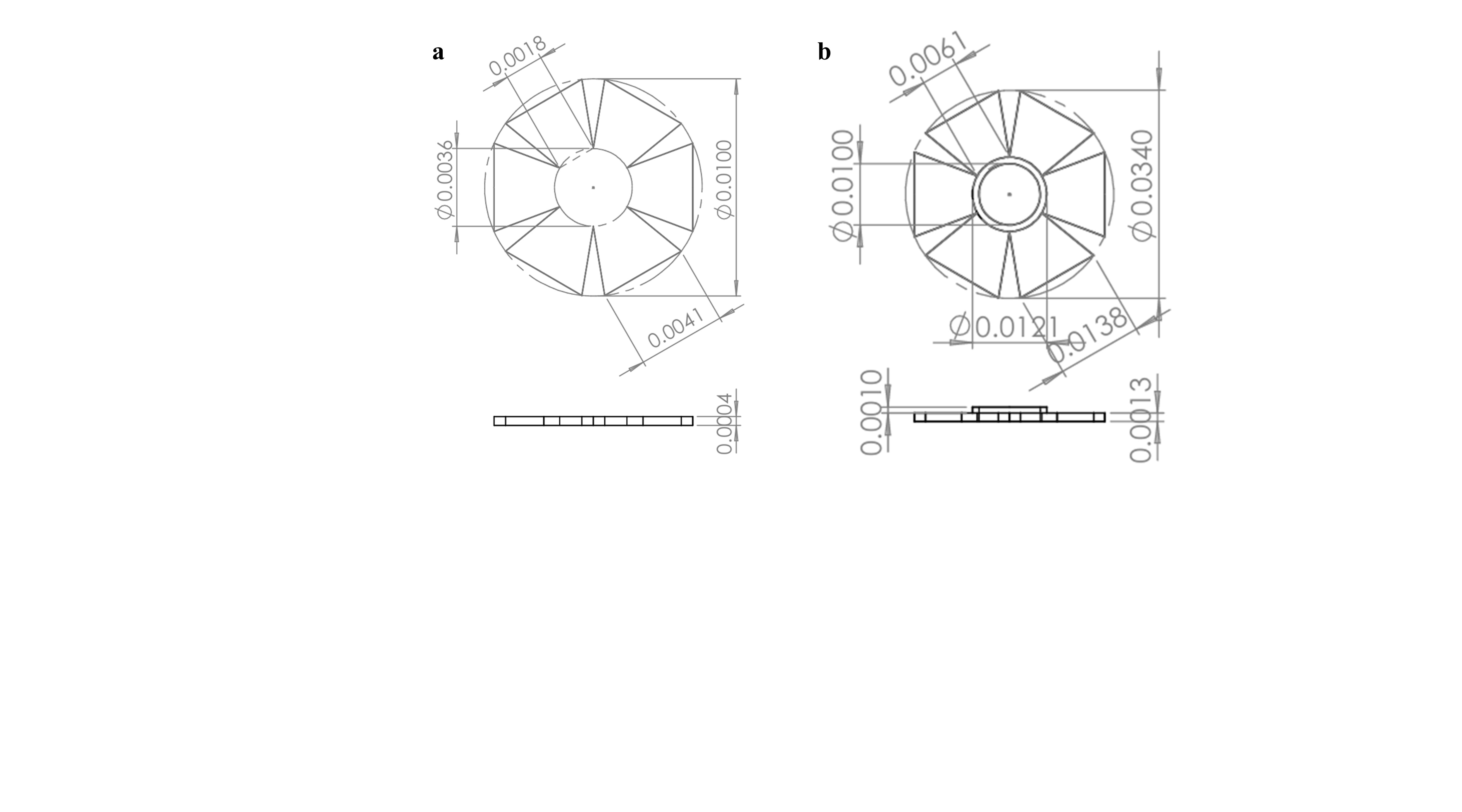


**Fig. S5. Dimension diagram of the J-MSR.** (a) Original J-MSR. (b) Expandable J-MSR (scaled up by 3.35 times than original size). All units are in meters**.**


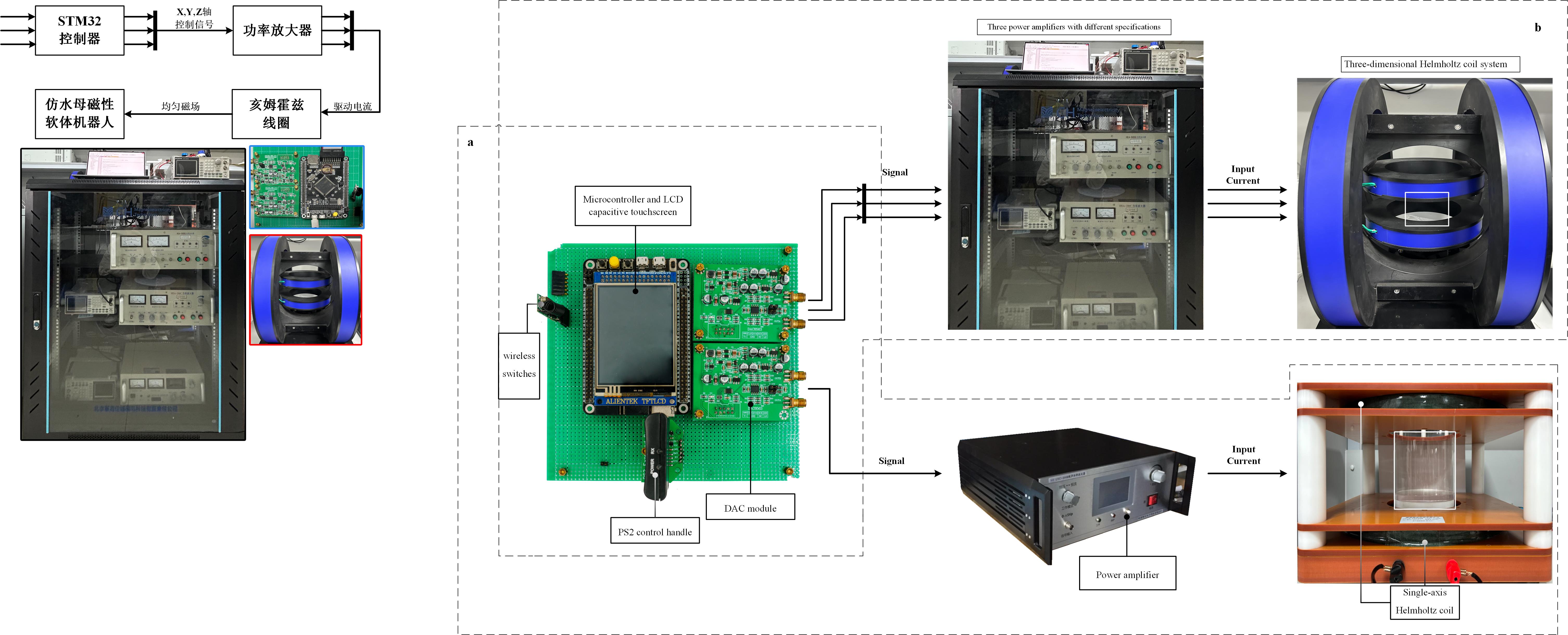


**Fig. S6.** **Schematic diagrams of 1D test platform and 3D manipulation platform.**


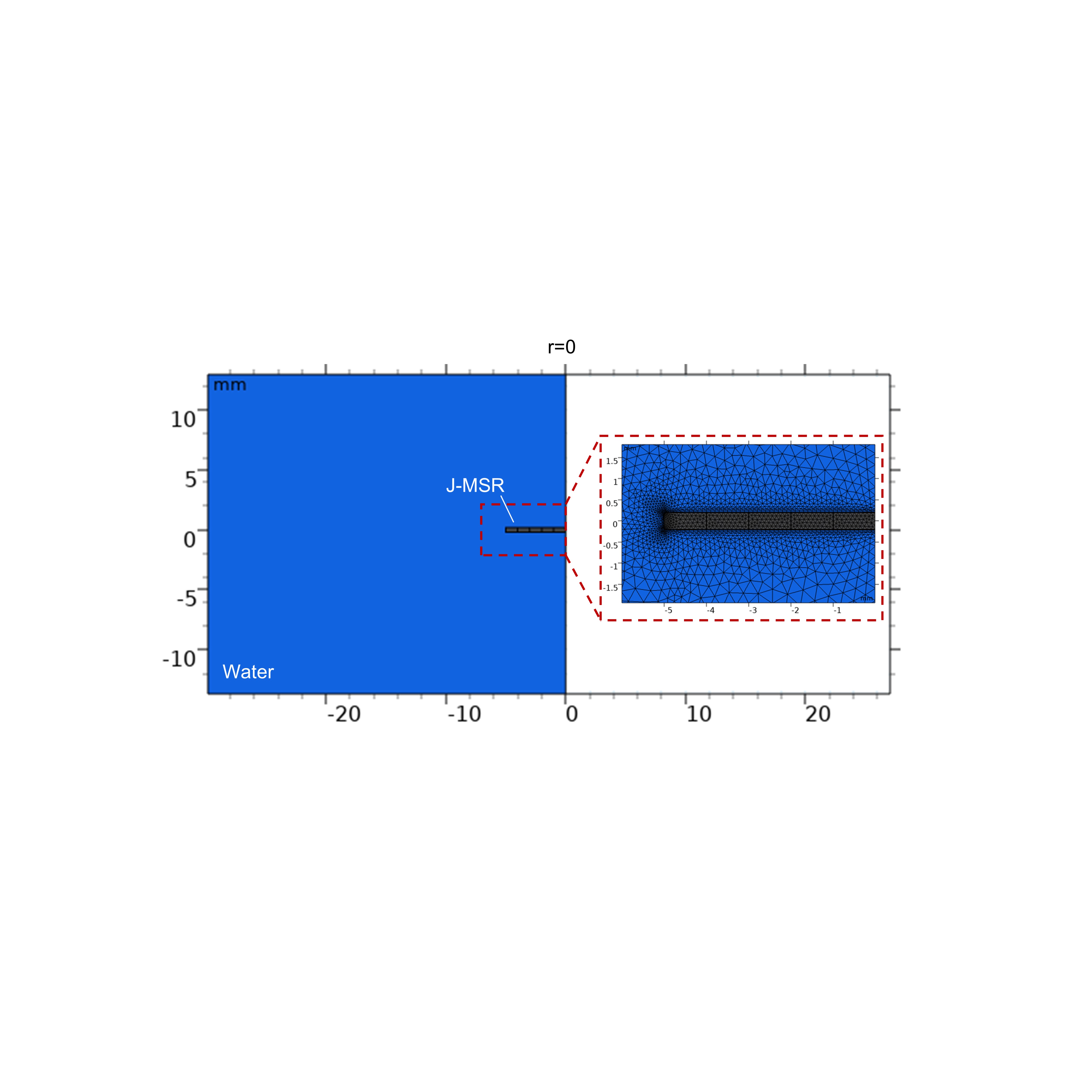


**Fig. S7.** **Finite element mesh of the J-MSR in simulations.**


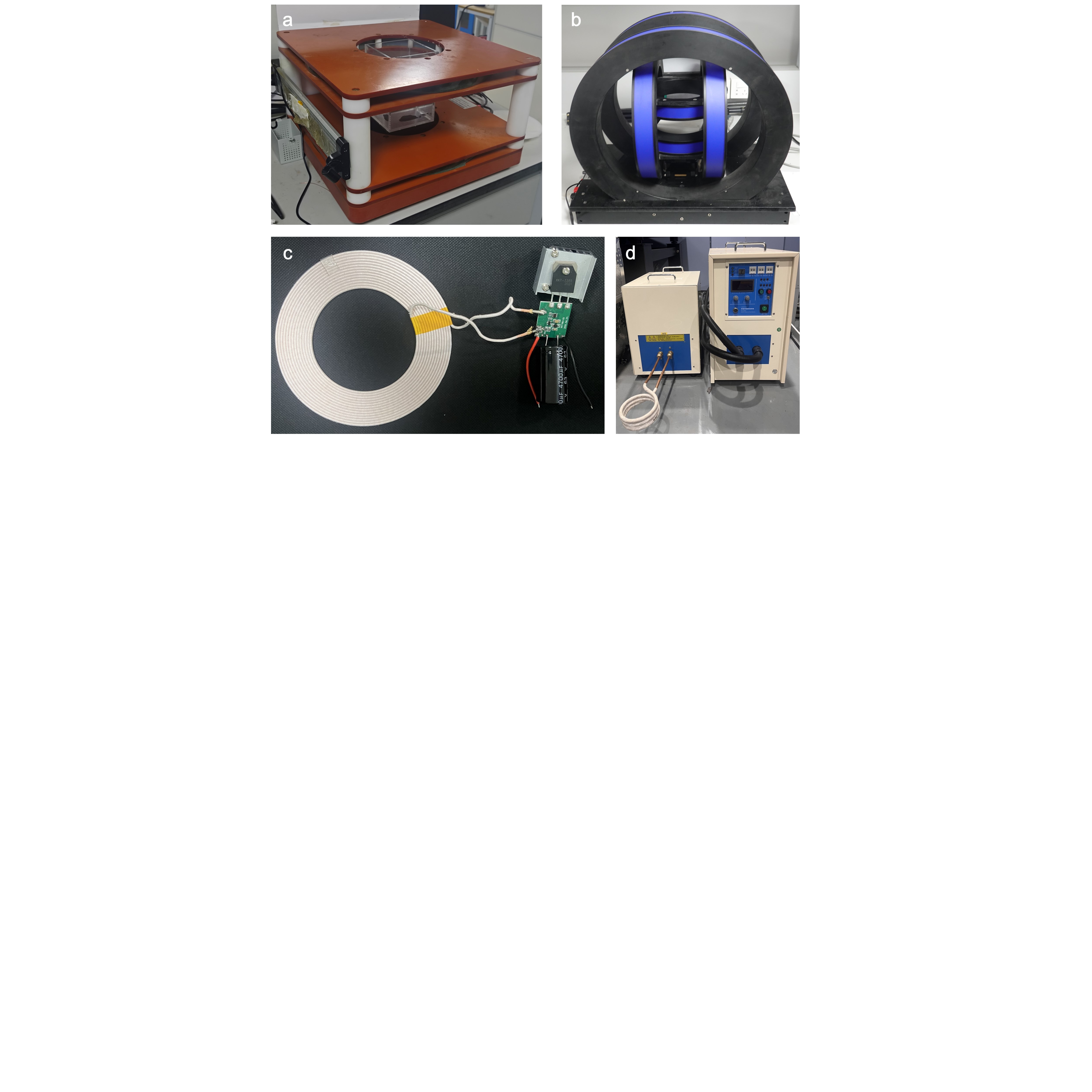


**Fig. S8.** **Pictures of magnetic field generating devices.** (a) Two-dimensional Helmholtz coils, (b) Three-dimensional Helmholtz coils, (c) Wireless powered transmitter Coil, (d) Induction heating machine.


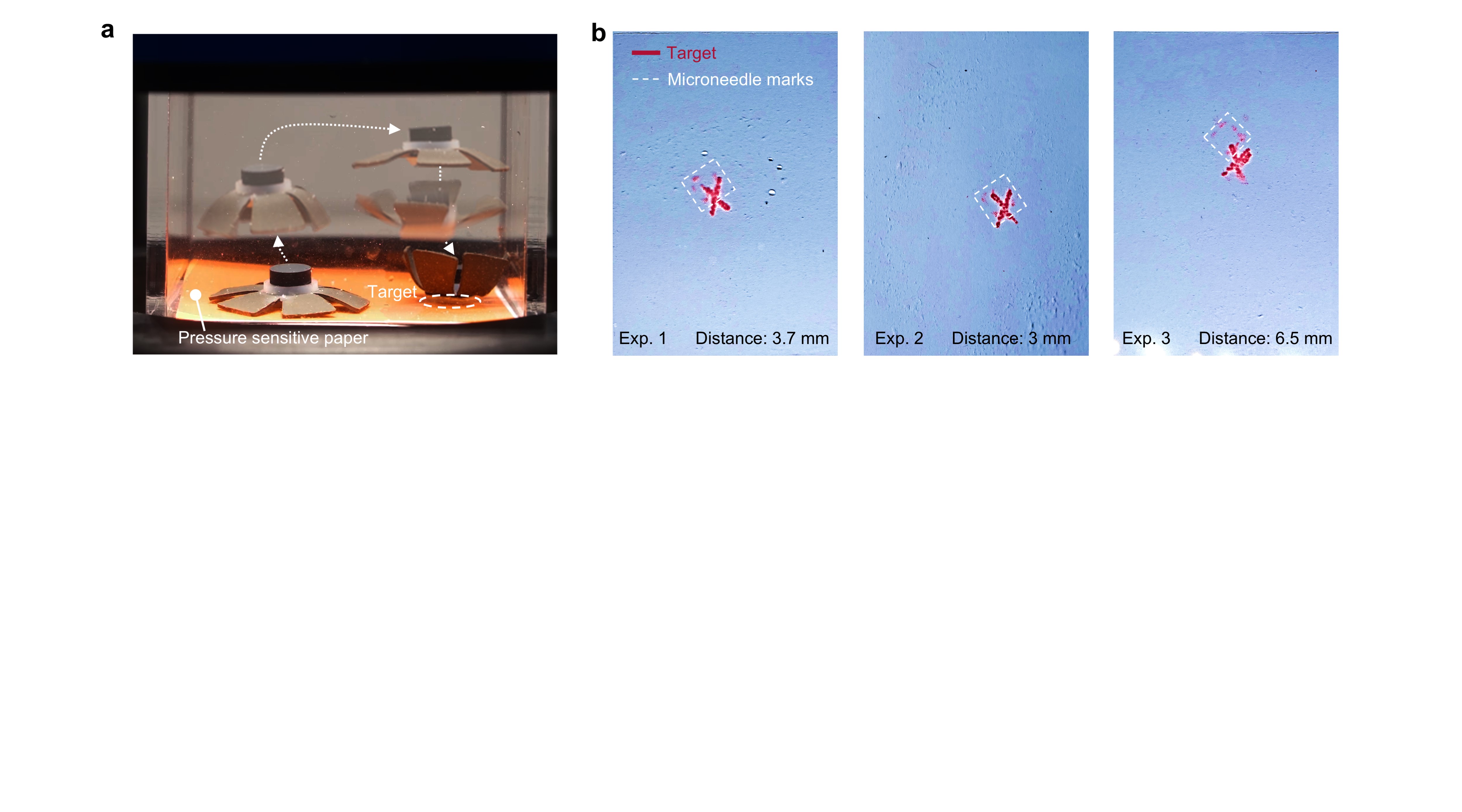


**Fig. S9. Targeting performance of J-MSR in a water tank under direct visual observation.** (a) Experimental setup and environment. (b) Pressure-sensitive paper records from three independent tests (n = 3).


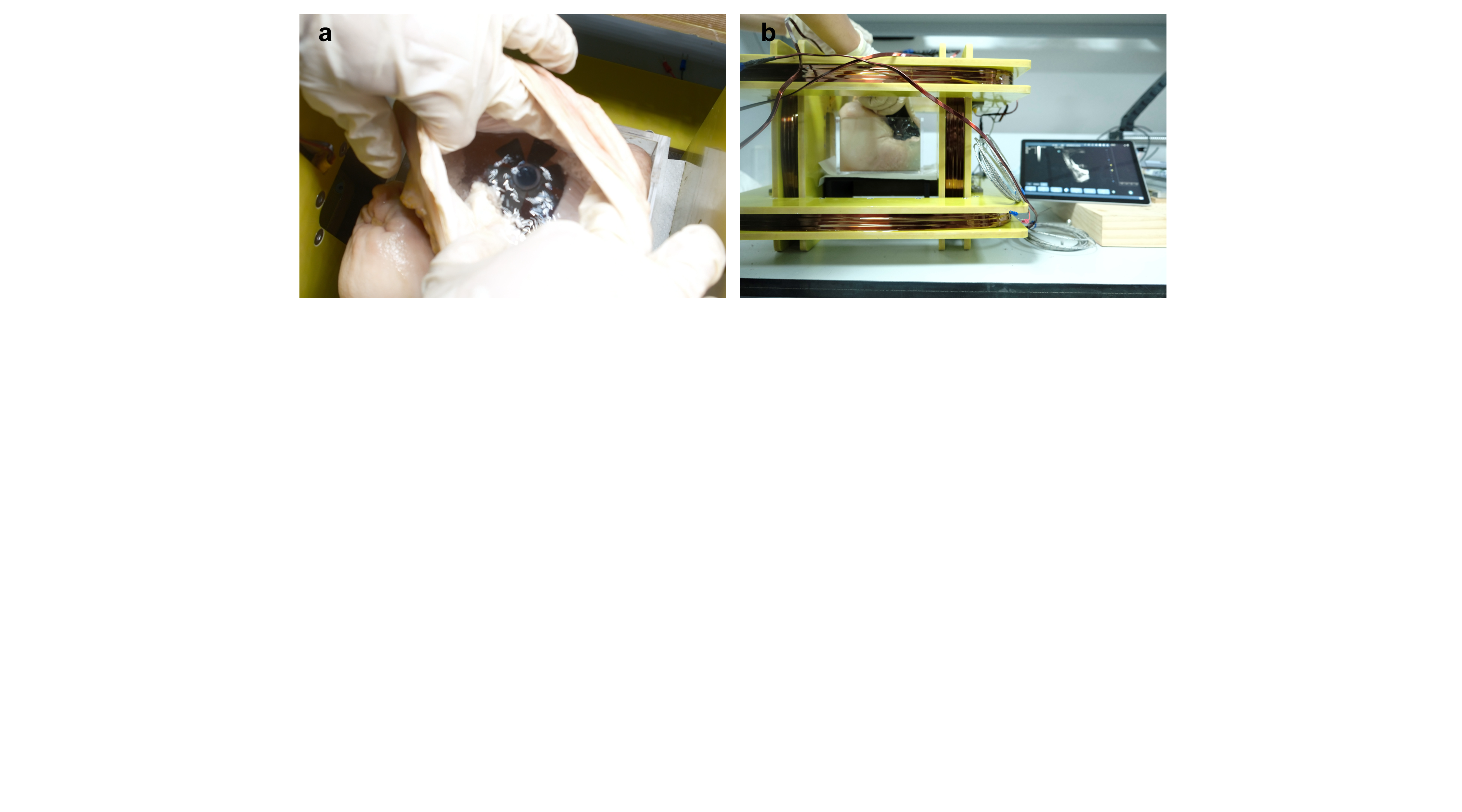


**Fig. S10.** **Experimental setup for ultrasound-guided targeting in an ex vivo porcine stomach.** (a) J-MSR placed inside the water-filled porcine stomach. (b) Porcine stomach positioned at the center of the square Helmholtz coil with the ultrasound probe attached.


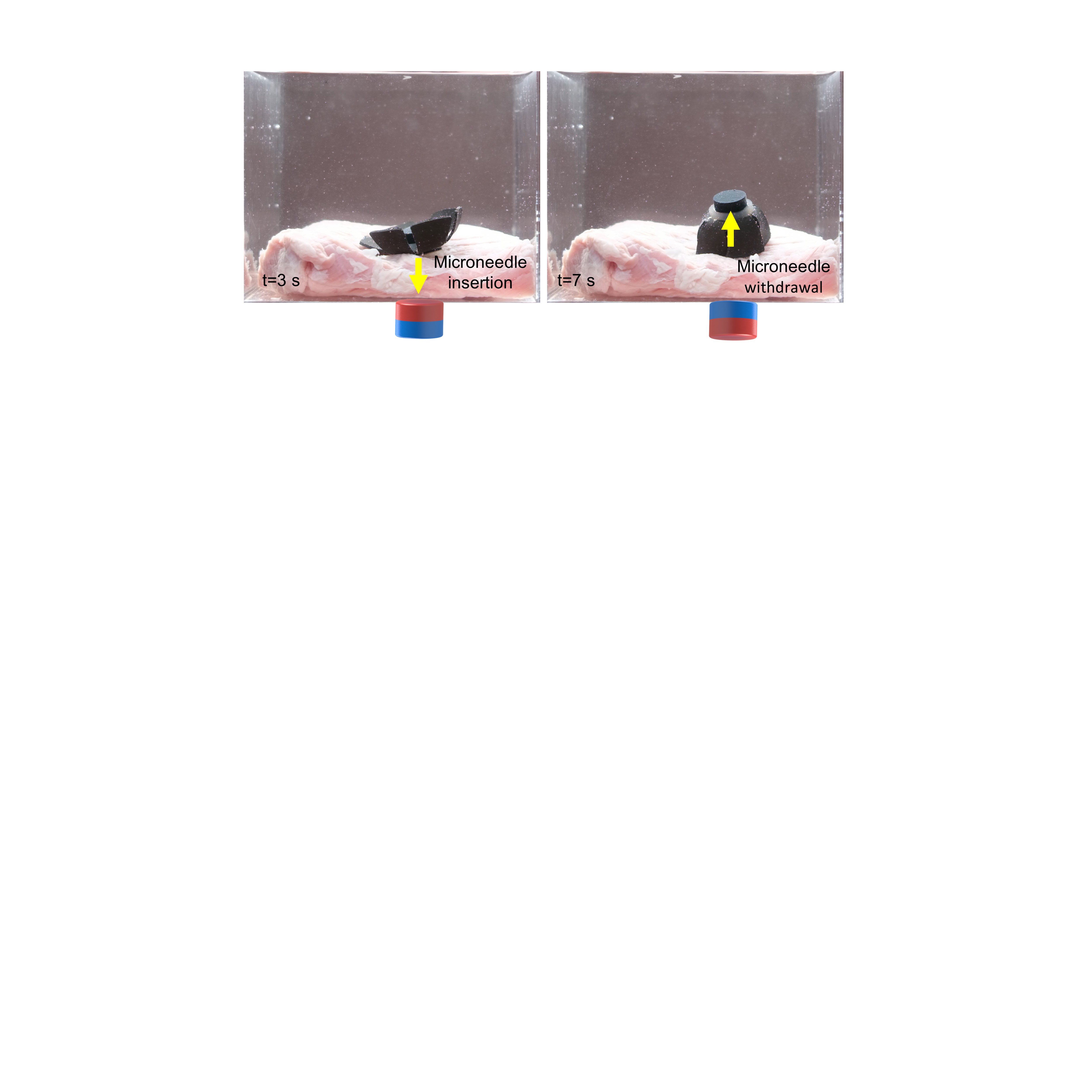


**Fig. S11. Reversible locomotion of the J-MSR enabled by magnetic field reversal.**


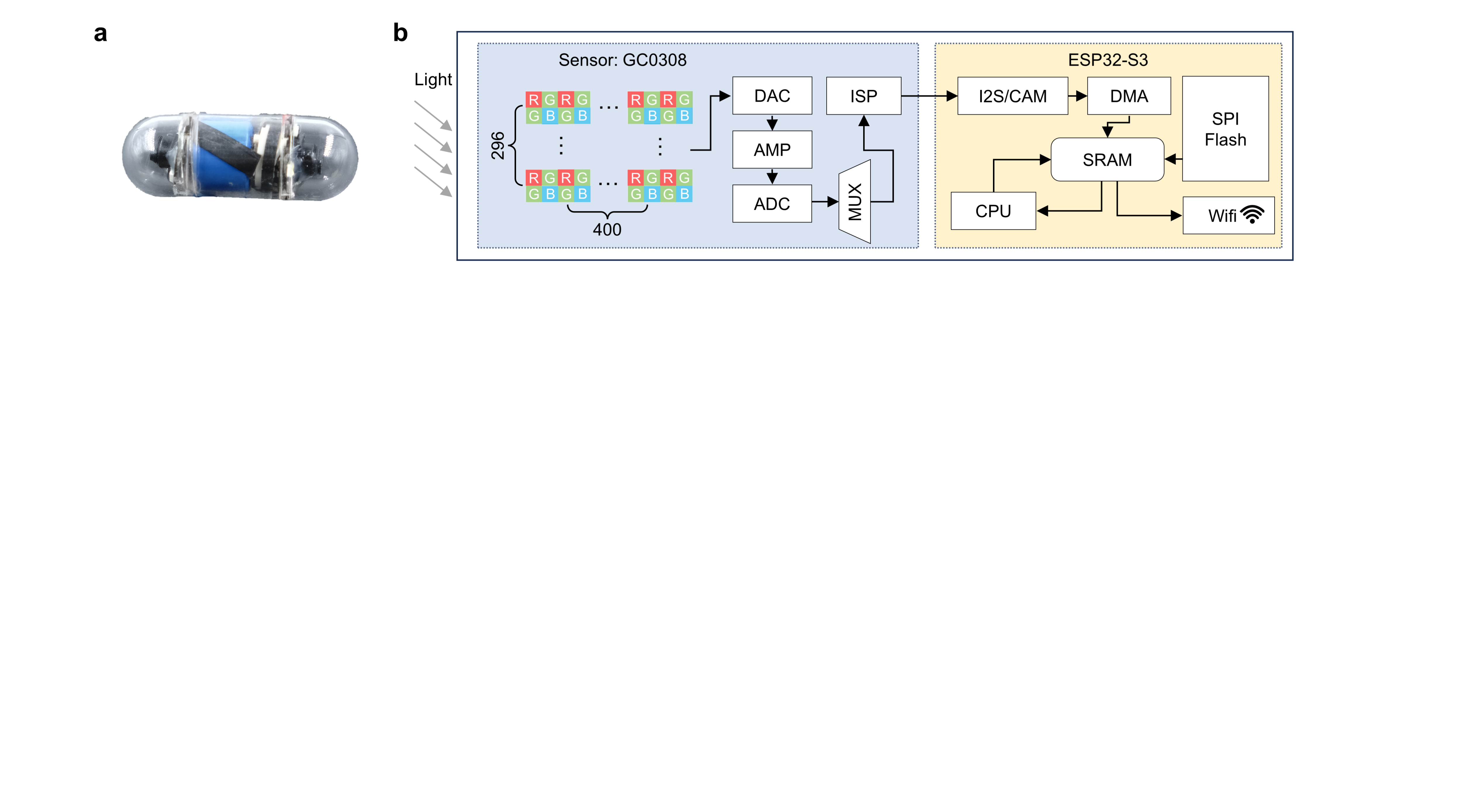


**Fig. S12**. **Design and system-level architecture of the binocular capsule endoscope integrated with the J-MSR.** (a) Photograph of the custom-designed binocular capsule endoscope. (b) System-level block diagram. DAC, digital to analogue converter; AMP, Amplifier; ADC, analogue to digital converter; MUX, multiplexer; ISP, image signal processor; I2S/CAM, inter-ic sound interface (camera interface); DMA, direct memory access controller; SRAM, static random access memory; SPI Flash, serial peripheral interface flash; CPU, Central Processing Unit.


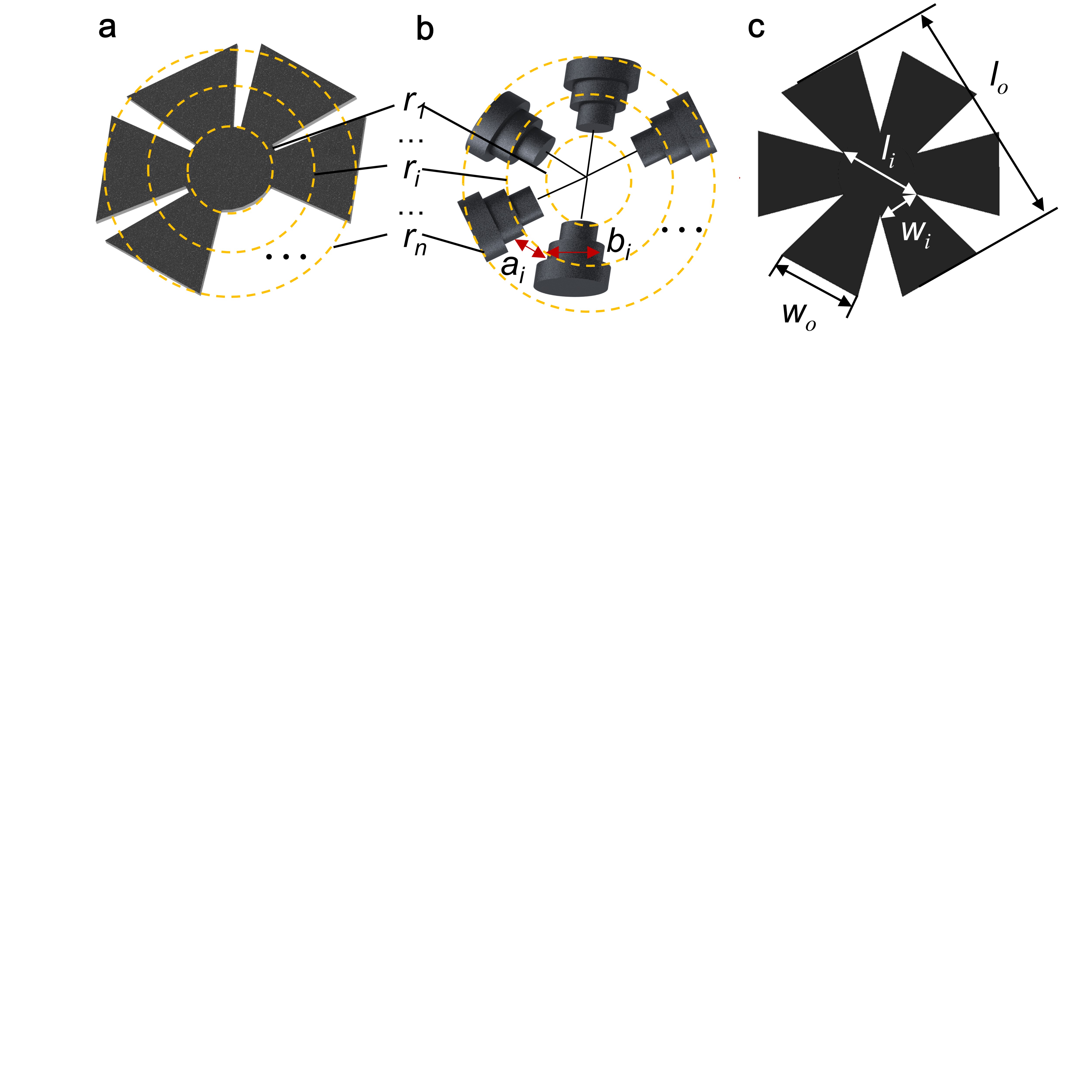


**Fig. S13.** **Schematic of the thrust analysis equivalent model.** (a) Original lappet array configuration before equivalence. (b) Equivalent cylindrical array representation. (c) Geometric parameters of the J-MSR.


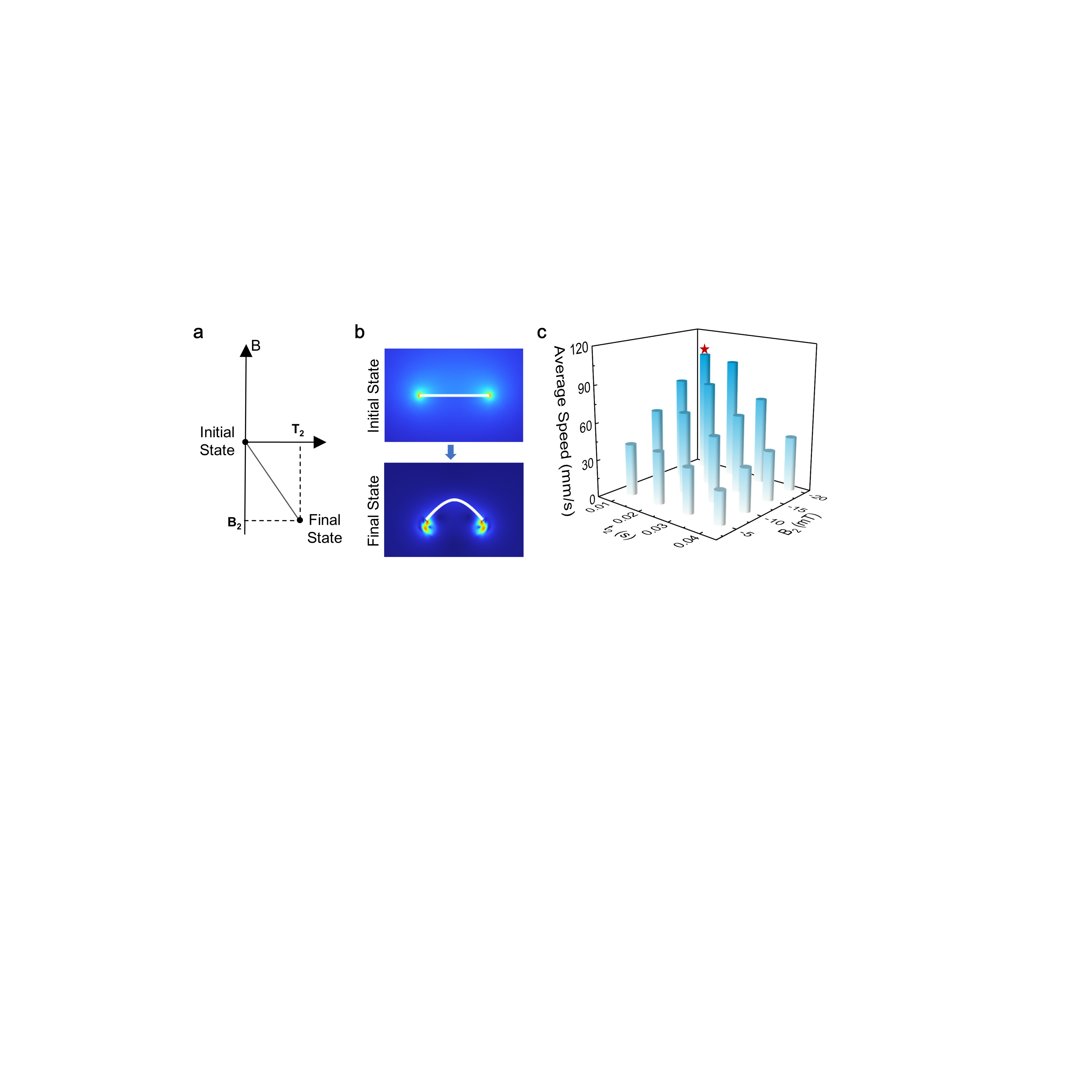


**Fig. S14.** **Simulation study on the settings of T_2_ and B_2_.** (a) the specific magnetic waveform configured to include only the contraction phase, (b) the initial and the final state of J-MSR during the simulation process. (c) distribution of average speeds under various combinations of contraction duration (T_2_) and reverse peak magnetic induction amplitude (B_2_).

**
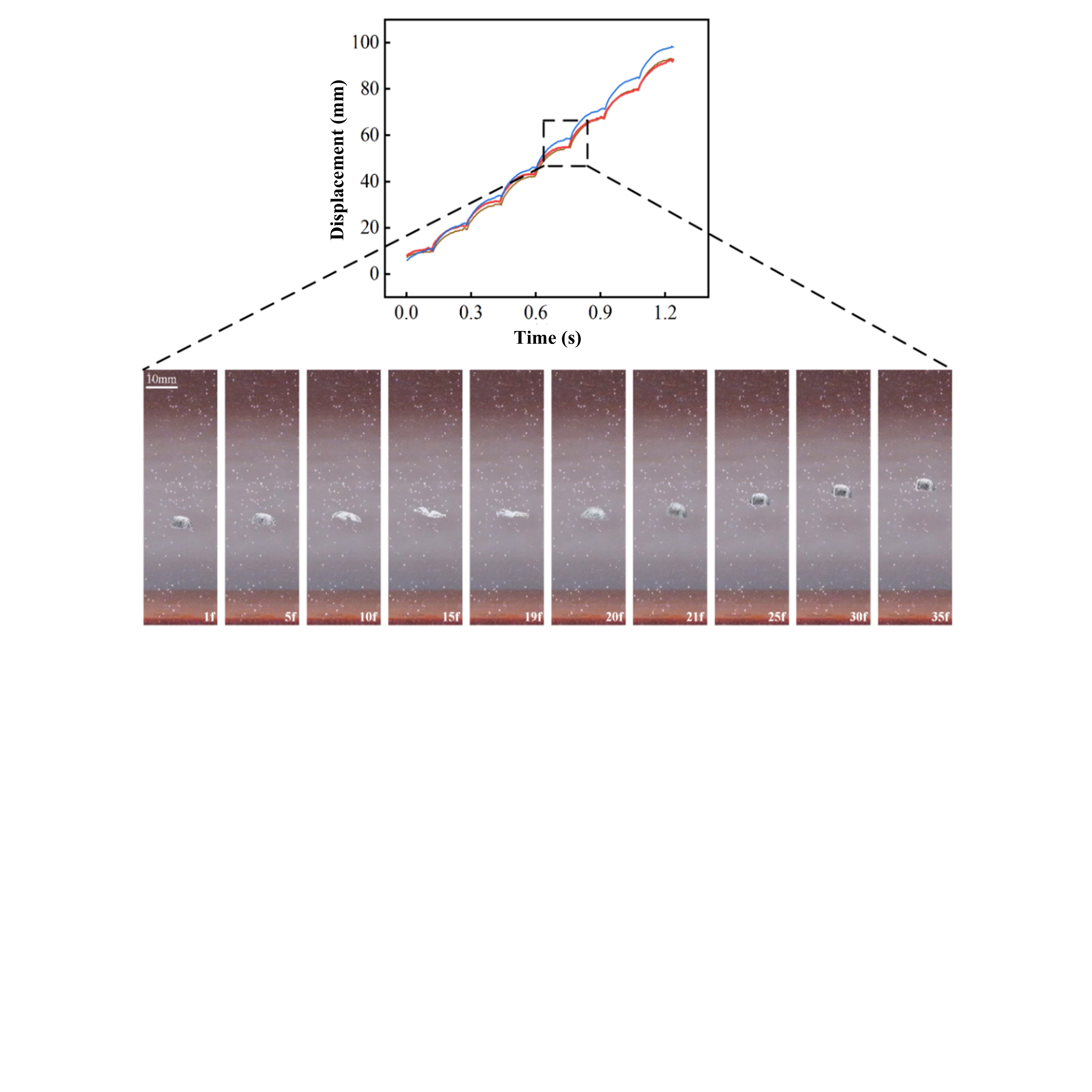
**

**Fig. S15. Displacement curve and images of the J-MSR’s motion process at B1=5mT and T1 =0.03s.**


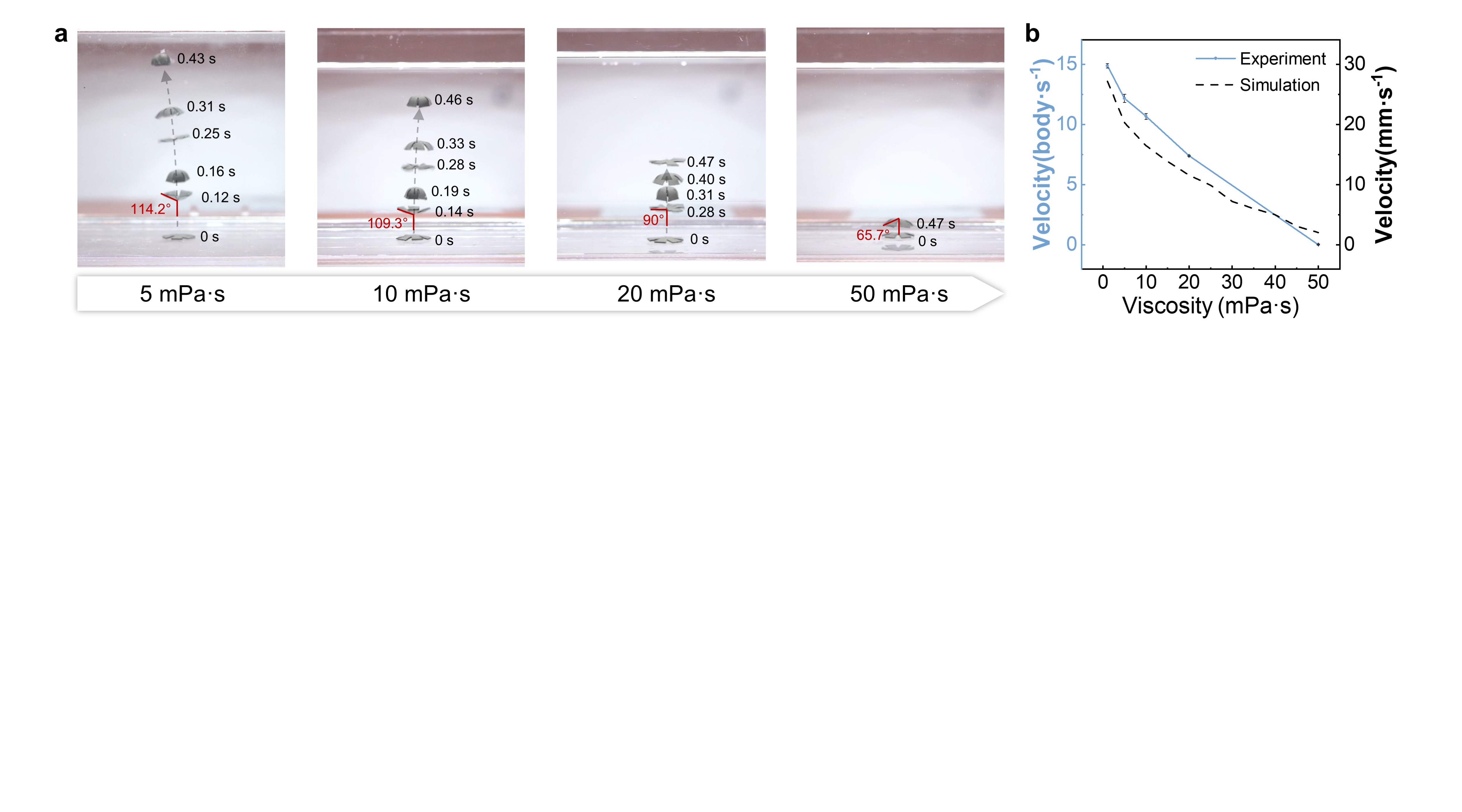


**Fig. S16. Effect of fluid viscosity on the swimming performance of the J-MSR.** (a) Swimming process of the J-MSR in fluids with different viscosities. (b) Swimming velocity of the J-MSR at different viscosities: experiment vs. simulation.


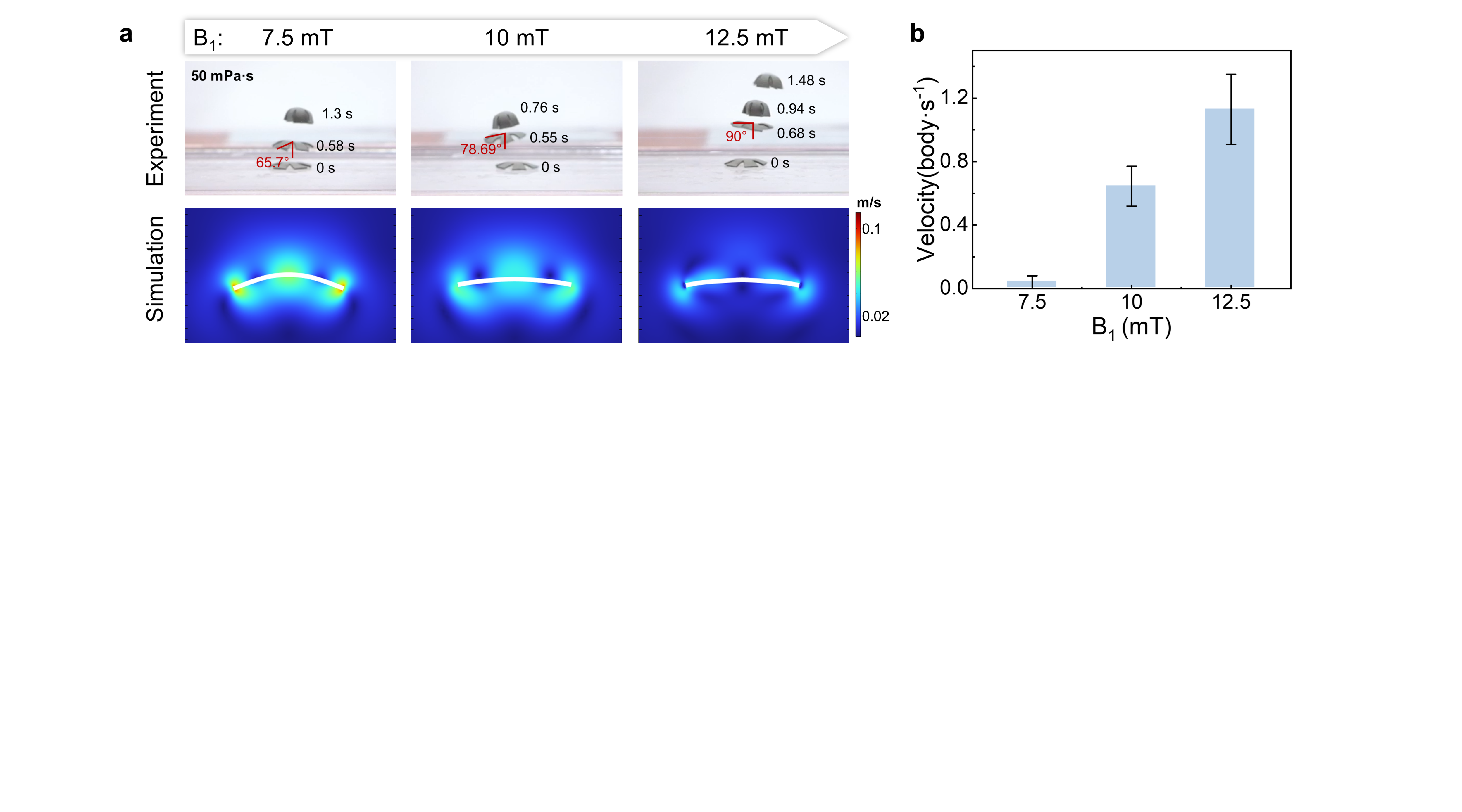


**Fig. S17. Improvement of J-MSR locomotion in high-viscosity fluids (50 mPa·s) by optimizing the actuation waveform.** (a) Locomotion process of the J-MSR in experiment and simulation under different B₁ values. (b) Swimming speeds of the J-MSR under different B₁ values in 50 mPa·s fluid.

**
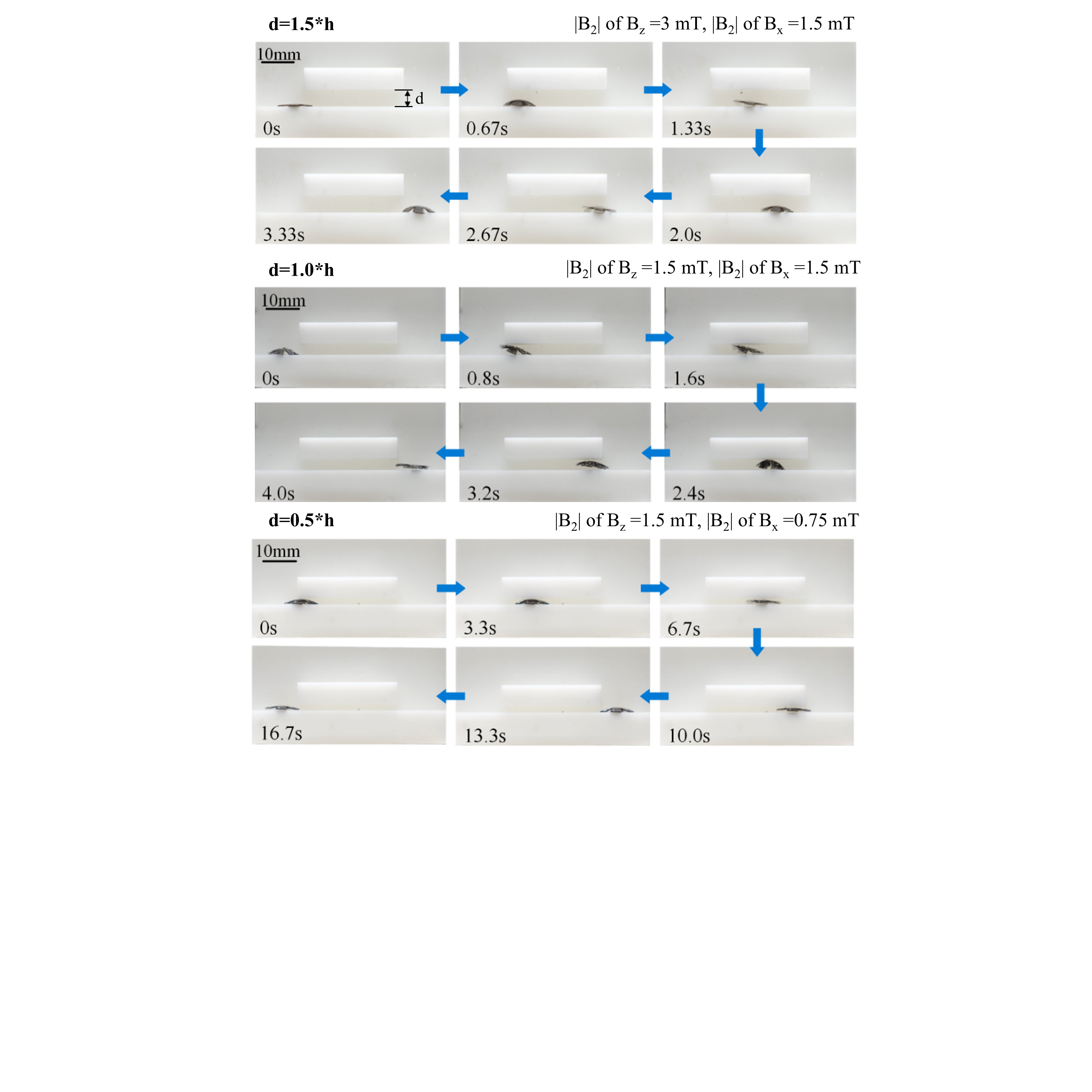
**

**Fig. S18. Images of the J-MSR's motion process as it crosses through slits of different heights.**


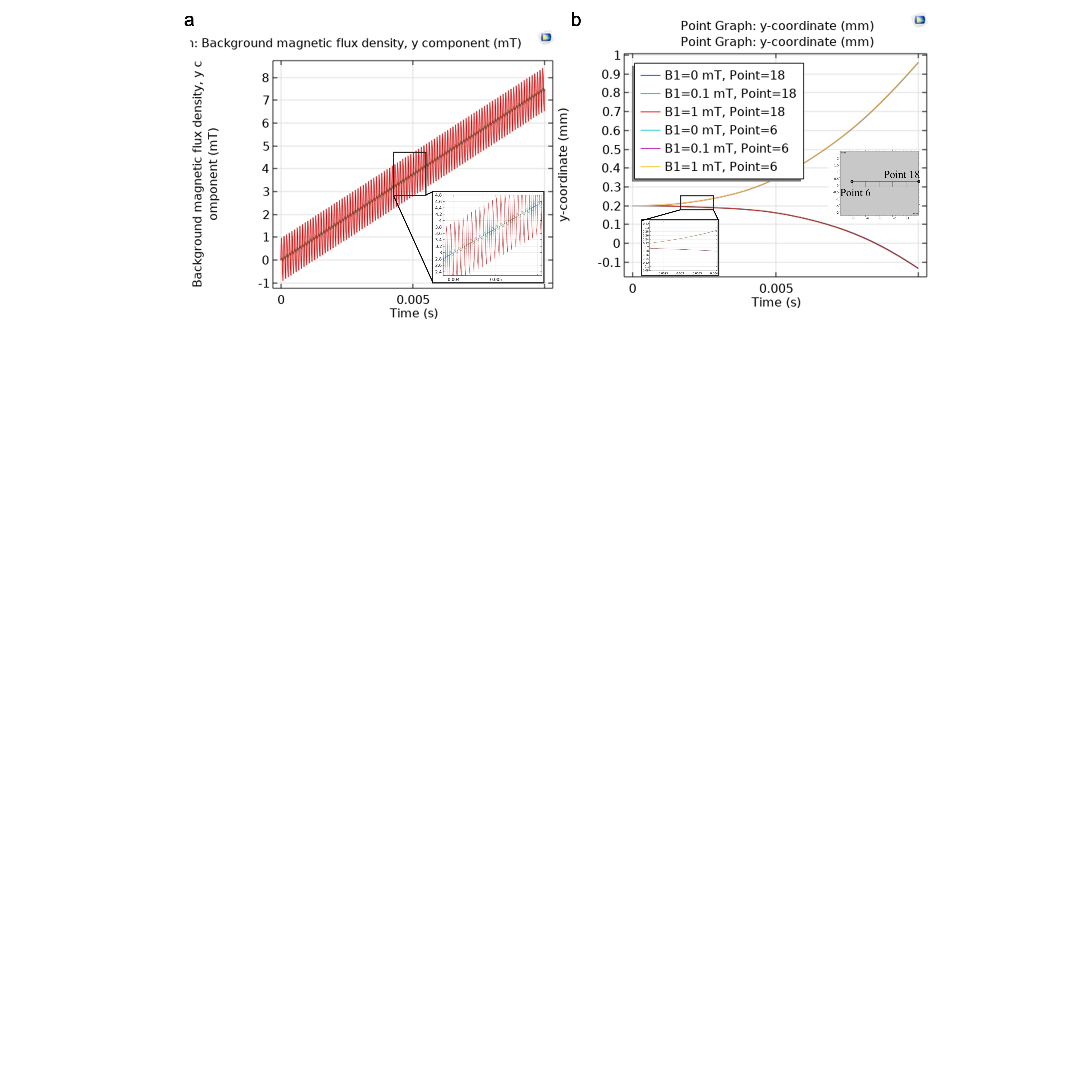


**Fig. S19. Dynamic response of the J-MSR under superimposed low- and high-frequency magnetic fields.** (a) Time profile of the superimposed background (low-frequency) and 10 kHz (high-frequency) magnetic fields at amplitudes 0.1 mT and 1 mT; (b) Displacement vs. time of the J-MSR centre and a lappet tip during the contraction phase, under the three high-frequency field amplitudes.

**Table S1. Comparison of the proposed jellyfish-inspired robots with the ones previously reported in the literature.**

| **References** | **Actuated Source** | **Density**  **(g/cm^3^)** | **Body length**  **(mm)** | **Speed**  **(BL/s)** | **3D motion** | **Catching & propulsion** | | **Expandable Functions** | **Driving waveform** | **Optimization variables** | **Simulation** | **Motion Mode** |
| --- | --- | --- | --- | --- | --- | --- | --- | --- | --- | --- | --- | --- |
| [3] | HASEL | 1.23* | 160 | 0.38125 | - | Yes | | Yes | Rectangular | Frequency, Duty cycle | Fluid-Solid (2D) | Swimming, Object transporting (via electrical control), Fluid mixing, Cooperative swimming |
| [4] | DE | -* | 156 | 0.0641 | No | No | | No | Rectangular | Frequency | Solid (3D) | Swimming |
| [5] | DE | -* | - | 0.2 | No | No | | No | Pulsed | Frequency | - | Swimming |
| [6] | DE&IPMC | 0.997* | 40 | 0.12 | **-** | No | | No | Sinusoidal | Frequency | - | Swimming |
| [7] | DE | 0.997* | 120 | 0.04166 | No | No | | No | Sawtooth | Frequency | - | Swimming |
| [8] | SMA | 1.1 | 141 | 0.14184 | No | No | | No | Rectangular | Input Power, Frequency | Solid (3D) | Swimming |
| [9] | SMA | 1.2 | 164 | 0.19 | No | No | | No | Pulsed and Rectangular | - | - | Swimming |
| [10] | Light | -* | 10 | 0.337 | Yes | No | | No | Rectangular | - | Fluid-Solid (3D, Fixed) | Swimming, Walking, Jumping |
| [11] | MF^1^ | -* | 100 | 0.06 | No | No | | Yes | Rectangular | Amplitude, Frequency, Duty cycle | Magnetic-Fluid-Solid (2D) | Swimming |
| [12] | MF | 1.02* | 5.75 | 4.05 | **-** | No | | Yes | Triangle, Trapezoidal | - | Magnetic-Fluid-Solid (2D, Fixed) | Swimming, Object transporting (via flow control), Burrowing, Fluid mixing |
| [13] | MF | -* | 50 | 4.6 | No | No | | No | Rectangular | Frequency, Bias rate | Solid (2D) | Swimming |
| [14] | MF | -* | 2.92 | 4.86 | Yes | No | | No | Triangle | - | - | Swimming |
| [15] | MF | -* | 9 | 0.8688 | - | No | | No | Triangle | - | - | Swimming, Rolling, Crawling |
| [16] | MF | 1.391 | 5 | 10 | Yes | No | | Yes | Trapezoidal | Amplitude, Frequency | - | Swimming, Object transporting (via flow control), Tube clogging |
| **Our work** | **MF** | **1.47** | **10** | **14.85** | **Yes** | **Yes** | | **Yes** | **Trapezoidal** | **B_1_, B_2_, T_1_, T_2_, T_3_, T_4_** | **Magnetic-Fluid-Solid (2D)** | **Swimming, Rolling, Crossing slit, Object transporting (via phase change), Load-carrying functional device (microneedles, Capsule endoscopy)** |
| HASEL: Hydraulically amplified self-healing electrostatic | | | | | | | MF: Magnetic field (magnetic torque) | | | | |  |
| DEA: Dielectric elastomer | | | | | | | 1: Lorentz force | | | | |  |
| IPMC: Ionic polymer metal composites | | | | | | | *: Buoyancy structure | | | | |  |
| SMA: Shape memory alloy | | | | | | |  | | | | |  |

**References:**

1. J. C. Nawroth, H. Lee, A. W. Feinberg, C. M. Ripplinger, M. L. McCain, A. Grosberg, J. O. Dabiri, K. K. Parker, A tissue-engineered jellyfish with biomimetic propulsion. *Nat Biotechnol* **30**, 792–797 (2012).
2. V. K. Satheesh, R. P. Chhabra, V. Eswaran, Steady incompressible fluid flow over a bundle of cylinders at moderate reynolds numbers. *The Canadian Journal of Chemical Engineering* **77**, 978–987 (1999).
3. T. Wang, H.-J. Joo, S. Song, W. Hu, C. Keplinger, M. Sitti, A versatile jellyfish-like robotic platform for effective underwater propulsion and manipulation. *Science Advances* **9**, eadg0292 (2023).
4. T. Cheng, G. Li, Y. Liang, M. Zhang, B. Liu, T.-W. Wong, J. Forman, M. Chen, G. Wang, Y. Tao, T. Li, Untethered soft robotic jellyfish. *Smart Mater. Struct.* **28**, 015019 (2018).
5. C. Christianson, C. Bayag, G. Li, S. Jadhav, A. Giri, C. Agba, T. Li, M. T. Tolley, Jellyfish-Inspired Soft Robot Driven by Fluid Electrode Dielectric Organic Robotic Actuators. *Front. Robot. AI* **6** (2019).
6. S. Wang, Z. Chen, Modeling of Two-Dimensionally Maneuverable Jellyfish-Inspired Robot Enabled by Multiple Soft Actuators. *IEEE/ASME Trans. Mechatron.*, 1–9 (2022).
7. S. Wang, Z. Chen, Modeling of jellyfish-inspired robot enabled by dielectric elastomer. *Int J Intell Robot Appl* **5**, 287–299 (2021).
8. M. A. A. Kazemi-Lari, A. D. Dostine, J. Zhang, A. S. Wineman, J. A. Shaw, Robotic jellyfish actuated with a shape memory alloy spring. *Bioinspiration, Biomimetics, and Bioreplication IX* **10965**, 1096504 (2019).
9. A. Villanueva, C. Smith, S. Priya, A biomimetic robotic jellyfish (Robojelly) actuated by shape memory alloy composite actuators. *Bioinspir. Biomim.* **6**, 036004 (2011).
10. C. Yin, F. Wei, S. Fu, Z. Zhai, Z. Ge, L. Yao, M. Jiang, M. Liu, Visible Light-Driven Jellyfish-like Miniature Swimming Soft Robot. *ACS Appl. Mater. Interfaces* **13**, 47147–47154 (2021).
11. J. Ye, Y.-C. Yao, J.-Y. Gao, S. Chen, P. Zhang, L. Sheng, J. Liu, LM-Jelly: Liquid Metal Enabled Biomimetic Robotic Jellyfish. *Soft Robotics*, soro.2021.0055 (2022).
12. Z. Ren, W. Hu, X. Dong, M. Sitti, Multi-functional soft-bodied jellyfish-like swimming. *Nat Commun* **10**, 2703 (2019).
13. Q. Wang, X. Lu, N. Yuan, P. Jiang, J. Yao, Y. Liu, J. Ding, Centimeter-Scale Underwater Robot with High-Speed Inspired by Jellyfish. *IEEE Robot Autom Let*, 1–7 (2023).
14. Y. Dai, S. Liang, Y. Chen, Y. Feng, D. Chen, B. Song, X. Bai, D. Zhang, L. Feng, F. Arai, Untethered Octopus‐Inspired Millirobot Actuated by Regular Tetrahedron Arranged Magnetic Field. *Advanced Intelligent Systems* **2**, 1900148 (2020).
15. C. Xu, Z. Yang, G. Z. Lum, Small-Scale Magnetic Actuators with Optimal Six Degrees-of-Freedom. *Advanced Materials* **33**, 2100170 (2021).
16. Z. Ren, T. Wang, W. Hu, M. Sitti, A Magnetically-Actuated Untethered Jellyfish-Inspired Soft Milliswimmer. *Robotics: Science and Systems*. 22-26 (2019).
